# Emergent “core communities” of microbes, meiofauna and macrofauna at hydrothermal vents

**DOI:** 10.1038/s43705-021-00031-1

**Published:** 2021-06-21

**Authors:** S. A. Murdock, V. Tunnicliffe, R. E. Boschen-Rose, S. K. Juniper

**Affiliations:** 1 School of Earth & Ocean Sciences, University of Victoria, Victoria, Canada; 2 Department of Biology, University of Victoria, Victoria, Canada; 3 Ocean & Earth Science, University of Southampton, Southampton, UK; 4 Ocean Networks Canada, University of Victoria, Victoria, Canada

**Keywords:** Water microbiology, Ecosystem ecology, Microbial ecology

## Abstract

Assessment of ecosystem health entails consideration of species interactions within and between size classes to determine their contributions to ecosystem function. Elucidating microbial involvement in these interactions requires tools to distil diverse microbial information down to relevant, manageable elements. We used covariance ratios (proportionality) between pairs of species and patterns of enrichment to identify “core communities” of likely interacting microbial (<64 µm), meiofaunal (64 µm to 1 mm) and macrofaunal (>1 mm) taxa within assemblages hosted by a foundation species, the hydrothermal vent tubeworm *Ridgeia piscesae*. Compared with samples from co-located hydrothermal fluids, microbial communities within *R. piscesae* assemblages are hotspots of taxonomic richness and are high in novelty (unclassified OTUs) and in relative abundance of Bacteroidetes. We also observed a robust temperature-driven distinction in assemblage composition above and below ~25 °C that spanned micro to macro size classes. The core high-temperature community included eight macro- and meiofaunal taxa and members of the Bacteroidetes and Epsilonbacteraeota, particularly the genera *Carboxylicivirga*, *Nitratifractor* and *Arcobacter*. The core low-temperature community included more meiofaunal species in addition to Alpha- and Gammaproteobacteria, and Actinobacteria. Inferred associations among high-temperature core community taxa suggest increased reliance on species interactions under more severe hydrothermal conditions. We propose refinement of species diversity to “core communities” as a tool to simplify investigations of relationships between taxonomic and functional diversity across domains and scales by narrowing the taxonomic scope.

## Introduction

From microbes to megafauna, interactions among species and with their environments shape biological assemblages [1] and, by extension, ecosystem function. Disentangling the outcomes of interactions can be a monumental task, particularly when attempting to include interactions involving the staggering diversity of microorganisms [2]. Growing recognition of the vital roles microorganisms play in marine ecosystems [3, 4] underscores the importance of integrating microbial and higher organism relationships when characterizing taxonomic and functional communities. A fundamental knowledge of the core players in these communities and how they interact can guide effective management strategies where human activities may compromise the ecosystem [5, 6].

A multi-scale approach to ecosystem characterization (i.e., one that incorporates multiple organismal size classes) can reveal covariances among organisms with direct links to shifts in ecosystem properties. For example, in marine sediments, multi-scale approaches revealed links between shifts in microbial abundance, faunal diversity, and ecosystem properties including biomass/productivity ratios, trophic structure, and nitrogen cycling [7–9]. In coastal ecosystems, the numerous and varied contributions of microphytobenthos to functionality [10] illustrate the importance of multi-scale interactions and feedbacks within ecological networks, and the vital roles of microbial players. However, moving from broad links between size classes to identifying species-level interactions requires methods for distiling the vast diversity of microbes down to those that are most likely interacting beyond the microbial realm.

One useful, first-order approach for identifying candidate multi-scale and interspecies associations is the analysis of co-occurrence patterns [11]. However, the use of co-occurrences to infer ecological interactions has been questioned [12], mainly because of inappropriate data handling and the potential for spurious correlations [13–15]. Proportionality, which considers covariance of species or OTU counts transformed to ratios [16], is an appropriate alternative for inferring associations. Covariance of microbial and faunal species may signal shared environmental preferences/tolerances or key inter-organism interactions (e.g., food webs, symbioses) and, therefore, be used to focus exploration of functional interactions on those species with the greatest potential importance to the ecosystem.

Hydrothermal vent ecosystems, where diverse non-photosynthetic prokaryotes are the exclusive primary producers, offer a potentially instructive case study for developing new approaches to integrating microbial and macrofaunal ecology. Microbial chemosynthetic primary production underpins the formation and maintenance of high-biomass faunal assemblages at low to moderate temperature (<100 °C, “diffuse”) hydrothermal vents [17] that result from mixing of high-temperature hydrothermal fluids and cold seawater within oceanic crust. These diffusively discharging hydrothermal fluids carry abundant microbes from subseafloor chemosynthetic primary production [18–20] and fuel additional chemosynthesis at and above the seafloor [21]. The resulting microbial biomass and metabolisms support grazing and deposit/suspension feeding animals and symbioses with animal partners [22]. Current understanding of hydrothermal vent fauna and microbes reflects more than four decades of research that has, with the exception of symbiotic associations, largely treated them as separate entities. Trophodynamics studies at vents have generalized food web links using stable isotope ratios [23–26] and lipid profiles [27–29] but have addressed neither the potential for incidental supply of organic material from fauna to support microbial heterotrophy nor the metabolic energy dynamics among microbes. Furthermore, these studies have not examined the composition or diversity of microbial food sources, only their biogeochemical signatures. Coordinated analyses of species-level variation in cohabiting vent fauna and microbes could be used to reveal potentially important interspecies associations beyond microbial-to-faunal food web links.

Habitats structured by foundation species increase physical stability and biodiversity [30–32] and, by extension, have greater potential for functional interactions across taxonomic groups. Siboglinid tubeworms, common foundation species in chemosynthesis-based vent habitats, physically augment access to chemosynthetic energy resources for microbes, and provide habitat structures for abundant associated fauna at density and biomass levels that rival other highly productive marine ecosystems (see review, [33]). Within tubeworm aggregations, the tube structures and their adhering microbial biofilms [34–36] slow the dispersion of hydrothermal fluids and so control physico-chemical gradients for other metazoa and microbes [37]. These broad co-dependencies provide direction for exploring potential functional relationships of micro- and macro-scale community members within the entire assemblage.

*Ridgeia piscesae* is a foundation siboglinid tubeworm species at vents on the mid-ocean ridges of the NE Pacific. The wide range of physico-chemical conditions occupied by *R. piscesae* [38] provides different habitats for associated faunal assemblages that vary in accordance with diffuse fluid flux and sulphide concentrations [39–41]. Non-endosymbiotic microbial composition across the tubeworm’s habitat range is less defined, although preliminary characterization spanning parts of this range suggests predictable patterns [42]. Full characterization of the microbiomes within these faunal assemblages also requires comparison with microbiomes of the hydrothermal fluids and seawater that flow through them. An integrated study of microbes and fauna in *Ridgeia*-hosted assemblages can advance understanding of how faunal assemblages contribute to augmenting microbial taxonomic diversity at vents—a relevant goal given the importance of biodiversity to ecosystem stability in the deep-sea [43]. Recent assessments of hydrothermal vent microbiomes reveal a tendency to consider only symbiotic microbes within faunal assemblages [44, 45], ignoring the potential selective or enriching effects of habitat conditions on non-endosymbiotic microbial composition within faunal assemblages.

Our objective is to develop and evaluate an approach for identifying “core communities” of likely interacting organisms within the diverse assemblages associated with hydrothermal vent tubeworm habitat. In particular, the study uses species-level diversity, enrichment and covariance to identify whether such a community transcends micro, meio, and macro size classes spanning a range of *R. piscesae* habitat conditions. Additionally, we assess tubeworm-assemblage microbial richness relative to corresponding hydrothermal fluids and background seawater to evaluate how faunal assemblages contribute to shaping microbial taxonomic diversity within the hydrothermal vent ecosystem. This detailed structural characterization of a hydrothermal vent microbiome represents an essential, yet nontrivial, first step toward identification of functional interactions with relevance to the ecosystem.

## Materials and methods

To remove ambiguity in the use of terms, we define the following: *assemblage*—cohabiting species whose level of interaction is unknown, *community*—cohabiting, covarying species that likely interact directly and may respond collectively to system changes. Methods are briefly described here with additional details in Supplementary Material.

### Sample collection

Microbial and faunal assemblages associated with the tubeworm *Ridgeia piscesae* (Supplementary Fig. 1) were sampled over a range of vent discharge intensities and supporting substrata (basalt and sulphide) using a manipulator arm on the remotely-operated vehicle ROPOS. These tubeworm grab samples came from vents on two segments of the Juan de Fuca Ridge (NE Pacific)—Endeavour (Main Endeavour & Clam Bed fields) and Middle Valley (Dead Dog and Bent Hill fields). Prior to collecting seven of the 13 samples, we used the ROPOS suction sampler to collect 2 Litres of hydrothermal fluids venting through the sampled tubeworm bushes. Background seawater was collected from each vent field in 5 L Niskin bottles. For all tubeworm grab samples, we first measured fluid temperatures inside the bush at the base. Details of sample location and habitat are in Table 1.

**Table 1:** Sample information.

			Location			Temperature (°C)		Balance of microbial domains^‡^			Observed faunal richness		
Sample name*	Sample Type	Substratum	Vent Field	Latitude (DecDeg)	Longitude (DecDeg)	Worm base	Fluid above worms	% Bacteria (± std err)	% Archaea (± std err)	% Microeukarya (± std err)	No. meiofaunal species	No. macrofaunal species	Total No. unique species†
**EMw1**	Tubeworm bush	Sulphide	Main Endeavour	47.9501	−129.0971	33.2		66.3 ± 2.6	27.3 ± 2.1	6.5 ± 0.5	5	7	11
EMw2	Tubeworm bush	Sulphide	Main Endeavour	47.9501	−129.0961	5.3		87.2 ± 0.2	8.2 ± <0.1	4.7 ± 0.2	na	na	na
**EMw3**	Tubeworm bush	Basalt	Main Endeavour	47.9500	−129.0971	3.4		82.1 ± 1.2	9.5 ± 0.4	8.4 ± 0.9	18	21	34
**EMw4**	Tubeworm bush	Sulphide	Main Endeavour	47.9500	−129.0970	37.1		98.2 ± <0.1	0.2 ± <0.1	1.6 ± <0.1	6	9	13
**EMw5**	Tubeworm bush	Sulphide	Main Endeavour	47.9500	−129.0969	13.4		83.4 ± 0.6	14.8 ± 0.6	1.8 ± <0.1	17	11	24
EMw6	Tubeworm bush	Basalt	Main Endeavour	47.9501	−129.0972	2.8		83.3 ± 0.7	4.8 ± 0.3	11.9 ± 0.8	na	na	na
EMw7	Tubeworm bush	Sulphide	Main Endeavour	47.9497	−129.0983	33.0^a^		99.0 ± <0.1	0.1 ± <0.1	0.9 ± <0.1	na	na	na
**EMw8**	Tubeworm bush	Sulphide	Main Endeavour	47.9495	−129.0985	37.4		95.9 ± 0.3	3.4 ± 0.3	0.7 ± <0.1	4	8	11
ECw9	Tubeworm bush	Sulphide	Clam Bed	47.9630	−129.0915	15.0		34.7 ± 0.3	63.2 ± 0.3	2.2 ± <0.1	na	na	na
**ECw10**	Tubeworm bush	Sulphide	Clam Bed	47.9630	−129.0915	31.0		95.5 ± <0.1	1.0 ± 0.1	3.5 ± 0.1	4	6	8
**ECw11**	Tubeworm bush	Basalt	Clam Bed	47.9631	−129.0917	5.2		93.9 ± <0.1	1.6 ± 0.1	4.5 ± 0.1	17	23	32
**MVw12**	Tubeworm bush	Sulphide	Middle Valley	48.4303	−128.6821	27.4		98.1 ± <0.1	0.8 ± <0.1	1.1 ± 0.1	1	5	6
**MVw13**	Tubeworm bush	Sulphide	Middle Valley	48.4553	−129.7086	21.1		84.5 ± 0.8	6.9 ± 0.8	8.5 ± <0.1	8	23	28
EMf1	Diffuse fluid	na	Main Endeavour	47.9501	−129.0971		5.0	92.0 ± 0.1	5.4 ± 0.1	2.6 ± 0.2			
EMf2	Diffuse fluid	na	Main Endeavour	47.9501	−129.0961		4.6	86.9 ± 0.7	3.1 ± 0.3	10.0 ± 0.4			
EMf3	Diffuse fluid	na	Main Endeavour	47.9500	−129.0971		4.1	75.2 ± 0.9	13.0 ± 0.3	11.8 ± 1.2			
EMf7	Diffuse fluid	na	Main Endeavour	47.9497	−129.0983		12.0	94.3 ± 0.3	1.4 ± <0.1	4.3 ± 0.3			
ECf9	Diffuse fluid	na	Clam Bed	47.9630	−129.0915		2.6	76.8 ± 0.7	12.5 ± 0.1	10.7 ± 0.6			
ECf11	Diffuse fluid	na	Clam Bed	47.9631	−129.0917		6.1	86.6 ± 0.6	7.0 ± 0.1	6.4 ± 0.6			
MVf12	Diffuse fluid	na	Middle Valley	48.4303	−128.6821		23.0	95.8 ± 0.2	3.3 ± 0.2	0.9 ± <0.1			
bkEC	Background fluid	na	Clam Bed	47.9631	−129.0915		na	75.9 ± 8.5	13.7 ± 3.5	10.4 ± 0.4			
bkEM	Background fluid	na	Main Endeavour	47.9501	−129.0972		na	74.4 ± 0.6	15.5 ± 0.6	10.1 ± 1.1			
bkMV	Background fluid	na	Middle Valley	48.4303	−128.6821		na	89.2 ± 9.5	4.6 ± 0.6	6.2 ± 0.2			

BOLD sample names were characterized from micro to macro.

*EM* endeavour-main, *EC e*ndeavour-clam bed, *MV* middle valley.

*Two letter codes denote sample location. Sample names with “f” or “w” correspond to diffuse fluids and tubeworm grabs, respectively. Numbers indicate the 13 sampled assemblages (i.e., EMw1 and EMf1 are associated with the same assemblage). Background fluid sample names begin with “bk”.

^‡^Determined by quantitative PCR.

†Unique means that juveniles of species occurring in the macrofaunal fraction were not included in this count.

Shipboard, approximately half of each tubeworm grab sample was placed in a bucket of cold (4 ˚C) artificial seawater and held at 4 ˚C until processing of the microbial component. The remaining half was preserved in either 75% ethanol or 7% buffered formalin (Supplementary Fig. 2). Later, tubeworms were removed from the preserved half, and the residue sieved to separate macrofauna (>1 mm) from meiofauna (>64 µm to <1 mm). See Supplementary Material for additional details of sample collection and faunal characterization. The microbial component associated with each grab sample was detached either by gently agitating the worm tubes in the bucket or, when possible, directly removing masses of adhering biofilm into a sterile 50 ml tube and agitating; thus, internal worm symbionts were not included. Two samples were subjected to both treatments to assess potential methodological bias (method replicates). For both methods, bucket water or biofilm was then passed through a 64 μm sieve to remove meiofauna and serially filtered onto 20 μm polycarbonate (micro size-fraction) and 0.2 μm Sterivex filters (pico/nano size-fraction) for DNA extraction. Cells from vent and background fluids were collected on 0.2 μm Sterivex filters onboard the ship using a peristaltic pump. All filters were stored at −80 °C until further processing.

### Microbial DNA extraction and sequencing

Following Sogin et al. [46], we extracted DNA from filtered cells associated with seven diffuse and three background fluid samples, and 13 tubeworm grab samples that included micro and pico/nano size fractions, plus two methodological replicates in each size fraction. Extractions included a sterile filter as a control. DNA quality and concentrations were measured on a NanoDrop 1000 (Thermo Scientific). Paired-end sequencing (2x300bp) of 16S/18S rRNA genes from 40 DNA extracts was completed on Illumina MiSeq at either the Laboratory for Advanced Genome Analysis (Vancouver Prostate Centre) or the Integrated Microbiome Resource Facility (Dalhousie University), using primers targeting Bacteria, Archaea, and microeukaryotes.

Paired-end sequence reads were merged and quality filtered using the *iu-merge-pairs* script from the Illumina-Utils package [47] with the *–enforce-Q30-check* flag and *min-qual-score* set to 20. Chimera removal, clustering into operational taxonomic units (OTUs), and taxonomic identification was performed using Mothur v1.42.3 [48] following the “MiSeq SOP” analysis example (https://mothur.org/wiki/MiSeq_SOP, accessed July 2019). Sequences were compared using the average neighbour pairwise distance method and clustered into OTUs using the standard 97% similarity threshold for bacteria and archaea and 98% for microeukaryotes [49]. Taxonomy was assigned to OTUs using the silva_nr_132 reference database [50] for bacteria and archaea and the PR2 v4.11.1 reference database [51] for microeukarya. Singleton OTUs, those with only one sequence in the entire dataset, were removed from further analysis.

### Quantitative PCR and microbial OTU scaling

Relative abundances of bacteria, archaea and microeukarya in each sample were determined by quantitative PCR (qPCR) of 16S and 18S rRNA genes. qPCR standards were created from a combination of clones for each microbial domain and used in tenfold dilution series to create standard curves. Sample reactions were performed in triplicate on a CFX96 Real-Time PCR Detection System (Bio-Rad), and each run included a standard curve and a no-template control. Samples were analyzed with DNA from the micro and pico/nano size fractions combined. Gene copies per ng of DNA were calculated based on concentrations of the DNA extracts.

OTU counts were converted to relative abundances within each domain and then combined and scaled according to the qPCR results. Each OTU was then expressed as abundance relative to all OTUs. This approximation of the natural microbial assemblage was subjected to the same tests as was each domain separately.

### Microbial composition and diversity

Data analysis followed the compositional approach as recommended by Gloor and Reid [15] and Quinn et al. [52]. Zero counts were replaced by imputed values using the count zero multiplicative method in the zCompositions R package [53, 54], and data were transformed by centred log-ratio [55]. To identify patterns in microbial assemblage composition using Analysis of Similarities (ANOSIM), hierarchical clustering and Nonmetric Multidimensional Scaling (NMDS), we calculated Aitchison distances between samples [56] using the coda.base R package [57].

Diversity calculations and compositional difference tests were performed using the vegan R package [58]. Diversity was calculated for each DNA extract and sequenced domain using the Inverse Simpson metric after singleton removal. The function ***metaMDS*** was used to create NMDS plots based on Aitchison distance matrices, and the ***envfit*** function was used to test for significant effects of temperature, substratum and location on NMDS ordinations. The ***hclust*** function was used for hierarchical cluster analysis.

ANOSIM was performed to identify potential biases introduced in our sampling methods and differences between samples related to environmental parameters or sample type. Details of bias tests are in Supplementary Material. Tests were performed on individual microbial domains and on the combined qPCR-balanced microbial assemblage. We compared compositions between samples from different substrata (sulphide, basalt), locations (Main Endeavour, Clam Bed, Middle Valley) and sample types (tubeworm grab, diffuse fluid, background fluid). OTUs responsible for significant compositional differences were identified using the ALDEx2 Bioconductor package v1.6.0 [59] and we report those with expected effect size differences ≥1, indicating relative enrichment in one category over the other.

### Size class congruence and taxonomic covariance

Using species and OTU counts for the macro, meio and micro size classes from nine grab samples, we explored covariance of species associated with *R. piscesae* across its range of habitat conditions. Microbes were assessed as both individual domains and the qPCR-balanced microbial assemblage. Prior to NMDS and hierarchical cluster analysis, macro- and meiofaunal species counts were treated with the same compositional data transformations as the microbial data (see above). Procrustes analysis (function ***procrustes***) on pairs of NMDS ordinations determined the level of congruence between size classes and microbial domains. Significance of the results was determined using the function ***protest*** with 1000 permutations. Both functions applied a symmetric analysis.

To identify covariance in ratios between microbial taxa and macro- and meiofaunal species, we performed proportionality analysis using a symmetric modification of the ρ metric in the propr R package [60]. Positive and negative ρ values between pairs of taxa indicate coincident occurrence or niche separation. Microbial OTUs with shared taxonomic identity (as assigned by Mothur) were aggregated into 719 unique taxonomic identities. To minimize the false discovery rate, taxa with counts of <2 in seven or more samples were excluded, leaving 335 microbial taxa, 14 macrofaunal and 17 meiofaunal species that were subjected to proportionality analysis. Taxon pairings with absolute ρ values > 0.75 were plotted using Cytoscape v3.7.2 [61].

## Results

### Composition and diversity

We produced 15.4 million (M) paired-end sequence reads of 16S and 18S rRNA genes from three microbial domains. Merged and quality filtered sequences consisted of 1.8 M bacterial, 2.7 M archaeal, and 2.3 M microeukaryal reads that clustered into 40,389 bacterial, 3436 archaeal, and 16,731 microeukaryal nonsingleton OTUs. Sample ECw11 produced <100 archaeal reads that were excluded from further analyses.

**Fig. 1:**
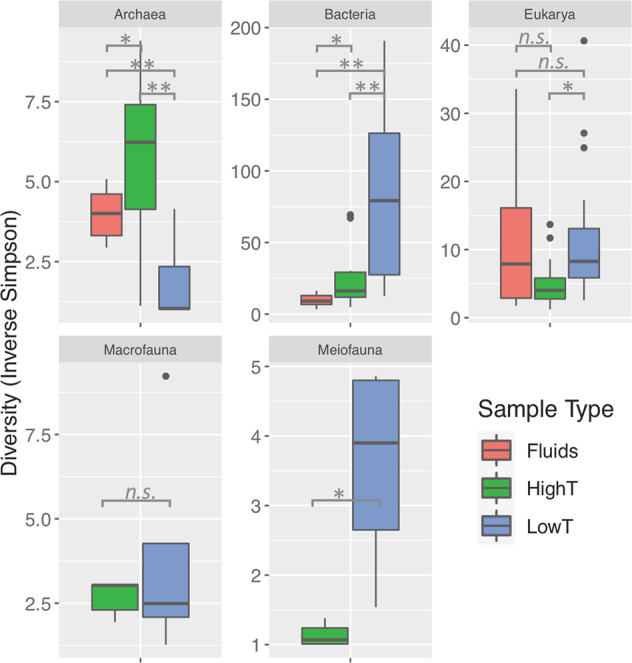
Inverse Simpson diversity of microbes (all 40 DNA extracts, post removal of singleton OTUs), macrofauna and meiofauna (nine grab samples). Grab samples are divided into “highT” and “lowT” (basal temperature above and below 25 °C, respectively). Significance values of Mann–Whitney tests: *p* < 0.01 (**), *p* < 0.05 (*), not significant (n.s.). Note varying y-axis scales.

Inverse Simpson diversity showed significant differences (Mann–Whitney tests) between microbes in fluid (background and diffuse) and grab samples; similarly, differences arose for microbes and fauna between grab samples with basal temperatures above and below *ca*. 25 °C, hereafter termed “highT grabs” (27–37°C) and “lowT grabs” (3–21 °C; Fig. 1). Bacterial diversity was greatest in lowT grabs and spanned a broad range of values, while in Archaea, diversity was greater in highT grabs. Microeukaryote and meiofaunal diversities were elevated in lowT versus highT grabs. Macrofaunal diversity did not differ between highT and lowT grabs (Fig. 1), although lowT samples had a wider range of diversity values, and highT showed consistent evenness values.

Bacteria dominated the microbial fraction in qPCR analysis (Table 1), with values ranging from 66 to 99% of the microbial 16S/18S copies for most samples. One anomalous lowT grab sample from Clam Bed (ECw9) was dominated by archaea (63% of 16S/18S copies). Otherwise, the average abundances of archaea and microeukarya were 7.2% and 5.8%, respectively, but ranged from below 1% up to 27% for archaea and near 12% for microeukarya. The balanced microbial assemblages reflecting these proportions were used for further analyses. For taxonomic breakdown of sequences before q-PCR balancing, see Supplementary Fig. 3.

Tests of potential biases on microbial composition introduced by our sample processing methods indicated minimal effects (see Supplementary Materials*)*. Data from the two size fractions from each sample were therefore combined, as were reads from the two harvesting methods for samples EMw1 and EMw6.

**Fig. 2:**
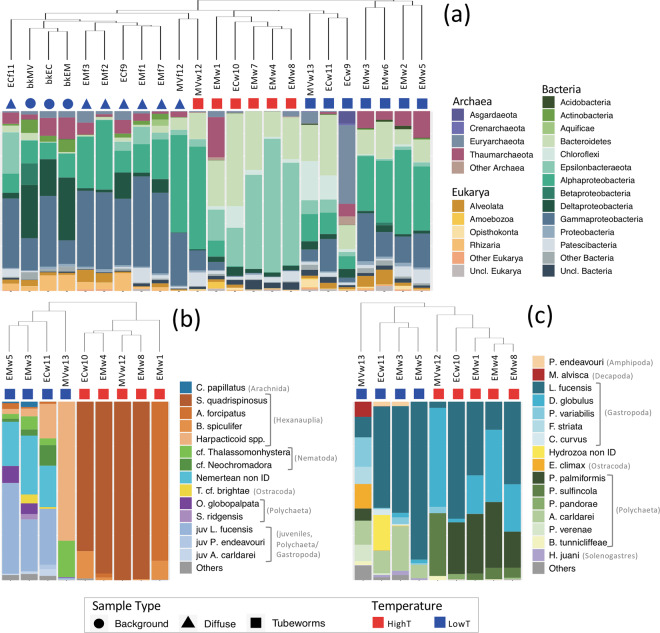
Composition of major groups of (${\mathbf{a}}$) microorganisms and (${\mathbf{b}}$) meio- and (${\mathbf{c}}$) macrofaunal species. Dendrograms show hierarchical clustering of samples based on Aitchison distances calculated from centred log-ratio transformed species and OTU counts. Sample types are indicated by coloured shapes—square=tubeworm grab; triangle=diffuse fluids; circle=background fluids—with shape colour indicating basal temperatures above (highT: red) or below (lowT: blue) 25 °C. “Other” includes taxa that were always <1% relative abundance in (**a**) and always <2% in (**b**, **c**). For major contributors within Gammaproteobacteria, Alphaproteobacteria, Bacteroidetes, and Epsilonbacteraeota, see *Supplementary Material* Fig. 4.

The qPCR-balanced microbial assemblages of diffuse fluid and background seawater samples grouped together in NMDS and hierarchical clustering analyses (Fig. 2a, Supplementary Fig. 4). Compositional differences between these two fluid types are well-known (e.g., [18, 62, 63]) and, therefore, are not discussed here. HighT grabs from Main Endeavour and Clam Bed clustered separately from lowT grabs. Among the tested environmental variables (temperature, substratum, location, sample type), only temperature explained variation in the NMDS (envfit *r*^2^ = 0.34, *p* = 0.017). NMDS ordinations of individual domains (not shown) revealed that the influence of temperature was strongest for archaea (envfit *r*^2^ = 0.60, *p* = 0.001) followed by bacteria (envfit *r*^2^ = 0.49, *p* = 0.001), whilst sample type (diffuse fluid, background fluid, tubeworm grab) emerged as a secondary predictor for archaea (envfit *r*^2^ = 0.45, *p* = 0.001). None of the tested environmental variables showed significant influence on microeukaryote composition.

ANOSIM tests run on individual domains returned similar results when using the “highT” and “lowT” designations ⎯ significant compositional differences based on temperature for archaea (*R* = 0.48, *p* = 0.001) and bacteria (*R* = 0.41, *p* = 0.015). There were no significant distinctions based on substratum, location or sample type, using either individual microbial domains or the balanced microbial assemblage.

Broadly, compositional differences between highT and lowT grabs occurred in four major bacterial groups (Fig. 2a). Epsilonbacteraeota and Bacteroidetes generally accounted for the majority of sequence reads in highT grabs and Gamma- and Alphaproteobacteria sequences were more numerous in lowT grabs. The highT grab from Middle Valley (MVw12) was the exception, being dominated by alphaproteobacterial sequences. For details of major contributors to these four bacterial groups, *see* Supplementary Fig. 5. Tubeworm grabs were primarily distinguished from fluids by greater numbers of Bacteroidetes sequences.

Tubeworm-associated fauna included 58 distinct macro- and meiofaunal taxa (Supplementary Tables 1, 2). Patterns of macro- and meiofaunal composition in NMDS ordinations were strongly influenced by temperature (Supplementary Fig. 6). Overall, dirivultid copepods comprised about 80% of the meiofauna with species identity shifting between highT and lowT; only lowT grabs returned nematodes and nemerteans (Fig. 2b). About 13% of overall meiofaunal numbers were juvenile macrofauna, although they were rare in highT grabs. Macrofaunal species abundances were heavily skewed with 90% of total counts represented by three species: two gastropods (*Lepetodrilus fucensis, Depressigyra globulus*) and one polychaete (*Paralvinella palmiformis*). The limpet, *L. fucensis* was the only species to occur in every grab sample. Macrofauna relative abundances were highly variable at lowT (Fig. 2c). The 21 °C Middle Valley sample returned ten species not found among Endeavour grabs. Combined faunal rarefaction (Supplementary Fig. 7) shows rapid levelling of species numbers at highT whereas two lowT grabs indicate larger samples would capture greater diversity. Further details of tubeworm-associated fauna are in Supplementary Material.

### Congruent variation between size classes and microbial domains

NMDS ordinations of macrofauna, meiofauna, and microbes (three domains, analyzed separately and combined) (Supplementary Fig. 6) showed strong Procrustes congruence between macro- and meiofauna (Table 2) followed by bacteria and microeukarya, then meiofauna with bacteria. The archaeal NMDS was not congruent with those of other microbial domains or size classes. Macro-meiofaunal relationships were consistently strong across samples, indicated by low Procrustes residual values (Supplementary Table 3), except one sample (MVw12). Although the combined microbial assemblage was not congruent with either macro- or meiofauna (Table 2), there was more congruence in the higher temperature samples (Supplementary Table 3). Similarly, Archaea showed some congruence with meiofauna in highT samples.

**Table 2:** Correlations between size classes and microbial domains determined by pairwise Procrustes analysis of NMDS plots.

	Macrofauna	Meiofauna	Bacteria	Archaea
Meiofauna	**0.86** (**0.26)**			
Bacteria	0.59 (0.65)	**0.63** (**0.61)**		
Archaea	0.60 (0.64)	0.38 (0.86)	0.32 (0.89)	
Microeukarya	0.31 (0.91)	0.47 (0.78)	**0.81 (0.34)**	0.22 (0.95)
Balanced microbes	0.42 (0.83)	0.52 (0.73)	–	–

Values indicate correlations (sum of squares). BOLD values are significant (*p*  <  0.05).

### Building communities

Potentially interacting taxa in highT and lowT *R. piscesae* habitats were identified using the combined results of enrichment and covariance (proportionality). In light of the strong temperature-driven distinction in faunal composition and congruence between macro- and meiofauna, we used highT- and lowT-enriched fauna as a starting point around which we built “core communities” of covarying taxa. Enriched meiofaunal species included distinct highT and lowT copepod associations and a nematode in only LowT (Supplementary Table 4). Among macrofauna, highT-enriched species included three alvinellid polychaetes, a polynoid polychaete and a snail, while at lowT ampharetid polychaetes and a solenogastre were relatively abundant.

**Fig. 3:**
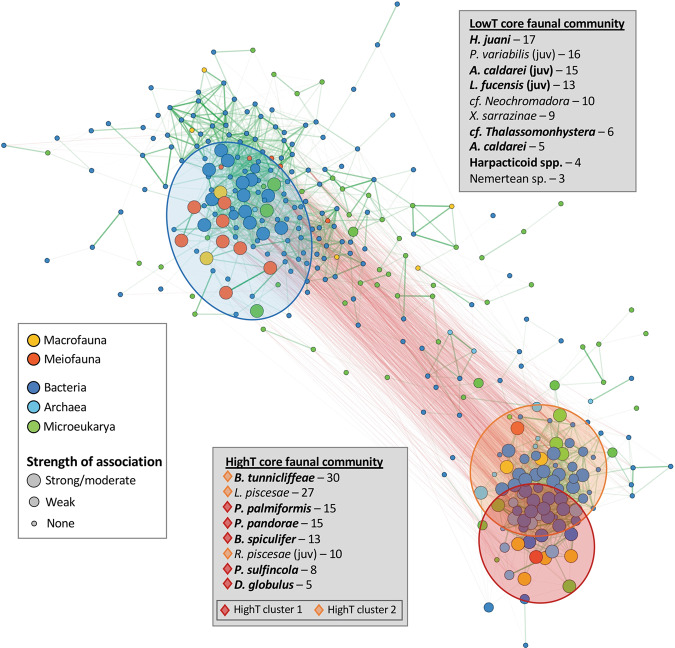
Co-occurrence network of invertebrates and microbes from nine tubeworm grab samples showing proportionality (ρ) values of >0.75 (green lines) or <−0.75 (red lines). Enriched faunal species (listed in **BOLD**) and taxa with strong to moderate associations to them comprise “core communities” and are indicated by large nodes. Taxa with weak associations (minimal covariance or enrichment only) are indicated by intermediate-sized nodes. HighT taxa formed two clusters (red and orange ellipses). Numbers after listed taxa indicate the total number of positive connections within the network. Details of large and intermediate node taxonomies can be found in Table 3 and Supplementary Table 5.

The 333 taxa included in proportionality analyses broadly separated into two clusters in the network diagram (Fig. 3), with faunal placement consistent with their enrichment in either highT or lowT grabs (see Supplementary Table 4). Taxa with no absolute ρ values ≥ 0.75 or with strictly/mostly negative ρ values were not included in the network diagram. Plotting only macro- and meiofauna (not shown) further partitioned highT-enriched species into clusters highT 1 and highT 2 (Fig. 3). Cluster 1 fauna had distinctly different relative abundances in highT and lowT samples while those in cluster 2 were more consistent over wider temperature ranges (Supplementary Fig. 8). LowT macro- and meiofauna had fewer overall network connections (9.8 ± 5.0) but more connections among faunal species (5.7 ± 2.2) relative to highT with 15.4 ± 8.8 overall connections and 1.5 ± 0.9 among-fauna connections. Enriched faunal species and co-varying taxa (proportionality *p* ≥ 0.75) with strong to moderate association with highT or lowT clusters (Table 3) are indicated by large nodes in the network diagram (Fig. 3) and considered members of core communities. Taxa with weaker associations (enrichment only or covariance with a single faunal species) with highT or lowT samples (Supplementary Table 5) are indicated as intermediate-sized nodes in Fig. 3 and considered as potentially transient community members.

**Table 3:** Microbial taxa with strong (covariance with fauna and enrichment) and moderate (covariance with multiple faunal species) associations with highT or lowT grab samples.

		Covariance (proportionality)				Relative enrichment^a^	
Supergroup/Phylum/ Class	Microbial taxa^b^	Cytoscape cluster^c^	Total no. positive ρ values	No. positive ρ with macro/meio	Average ρ with macro/meio	vs. LowT grabs	vs. HighT diffuse
***Strong associations with highT grabs***							
Bacteroidetes	B_Carboxylicivirga	HighT 1/2	39	5	0.81	X	X
Epsilonbacteraeota	B_Nitratifractor	HighT 1	34	3	0.81	X	X
Epsilonbacteraeota	B_Arcobacter	HighT 1	28	2	0.83	X	X
Bacteroidetes	B_cl_Bacteroidetes VC2.1 Bac22	HighT 2	43	1	0.77	X	X
Bacteroidetes	B_or_Bacteroidales BD2-2	HighT 2	34	1	0.77	X	X
Bacteroidetes	B_or_Sphingobacteriales	HighT 2	26	1	0.75	X	X
Asgardaeota	A_sg_Asgardaeota	HighT 2	13	1	0.76	X	
Bacteroidetes	B_fa_Lentimicrobiaceae	HighT 2	45	2	0.78	X	
Bacteroidetes	B_Ichthyobacterium	HighT 1	17	2	0.80	X	
Bacteroidetes	B_Maritimimonas	HighT 2	27	1	0.76	X	
Deltaproteobacteria	B_fa_Desulfobulbaceae	HighT 2	40	2	0.77	X	
Epsilonbacteraeota	B_Hydrogenimonas	HighT 2	30	1	0.87	X	
Epsilonbacteraeota	B_or_Campylobacterales	HighT 1	44	1	0.77	X	
Excavata	E_or_Jakobida	HighT 1/2	24	2	0.82		X
Opisthokonta	E_or_Pezizomycotina	HighT 2	9	1	0.78		X
***Moderate associations with highT grabs***							
	A_do_Archaea	HighT 1/2	33	3	0.81		
Euryarchaeota	A_fa_Aciduliprofundaceae	HighT 1	25	3	0.81		
Euryarchaeota	A_Methanocaldococcus	HighT 1	33	2	0.85		
Euryarchaeota	A_Thermococcus	HighT 1/2	31	4	0.81		
Aquificae	B_fa_Desulfurobacteriaceae	HighT 1	24	2	0.81		
Aquificae	B_Thermosulfidibacter	HighT 2	14	3	0.84		
Bacteroidetes	B_Draconibacterium	HighT 1/2	40	5	0.80		
Bacteroidetes	B_fa_Marinilabiliaceae	HighT 1/2	48	5	0.81		
Bacteroidetes	B_or_Bacteroidales	HighT 2	35	2	0.83		
Calditrichaeota	B_Calorithrix	HighT 2	30	3	0.86		
Deltaproteobacteria	B_Desulfuromonas	HighT 1	30	3	0.85		
Deltaproteobacteria	B_Dissulfurimicrobium	HighT 2	49	2	0.87		
Deltaproteobacteria	B_fa_Desulfuromonadaceae	HighT 1/2	36	4	0.82		
Deltaproteobacteria	B_or_Desulfobacterales	HighT 2	36	3	0.82		
Epsilonbacteraeota	B_fa_Nitratiruptoraceae	HighT 1/2	43	5	0.82		
Epsilonbacteraeota	B_Nitratiruptor	HighT 1/2	41	3	0.82		
Thermodesulfobacteria	B_fa_Thermodesulfobacteriaceae	HighT 2	42	2	0.86		
Amoebozoa	E_Vermistella vermiformis	HighT 1	5	2	0.78		
Opisthokonta	E_fa_Dothideomycetes	HighT 2	6	2	0.77		
Opisthokonta	E_fa_Eurotiomycetes	HighT 2	11	2	0.86		
***Strong associations with lowT grabs***						vs. HighT grabs	vs. LowT diffuse
Actinobacteria	B_fa_Sva0996 marine group	LowT	23	4	0.79	X	X
Gammaproteobacteria	B_Halioglobus	LowT	30	3	0.79	X	X
Alphaproteobacteria	B_fa_Devosiaceae	LowT	16	1	0.78	X	X
Bacteroidetes	B_Euzebyella	LowT	25	1	0.79	X	X
Cyanobacteria	B_cl_Sericytochromatia	LowT	23	1	0.76	X	X
Gammaproteobacteria	B_Dasania	LowT	13	1	0.79		X
***Moderate associations with lowT grabs***							
Actinobacteria	B_cl_Acidimicrobiia	LowT	14	2	0.83		
Actinobacteria	B_Iamia	LowT	40	3	0.81		
Cand. Peregrinibacteria	B_cl_Candidatus Peribacteria	LowT	34	3	0.79		
Deltaproteobacteria	B_fa_Oligoflexaceae	LowT	27	3	0.78		
Deltaproteobacteria	B_Nannocystis	LowT	30	3	0.80		
Gammaproteobacteria	B_Marimicrobium	LowT	29	3	0.79		
Gammaproteobacteria	B_or_Cellvibrionales	LowT	40	3	0.77		
Cercozoa	E_Thalassomyxa lineage	LowT	23	2	0.81		
Ciliophora	E_Holosticha	LowT	28	6	0.83		
Ciliophora	E_or_Hypotrichia	LowT	5	3	0.80		

^a^Relative enrichment determined by ALDEx2 pairwise tests.

^b^Multiple OTUs binned by identical taxonomic assignment. Taxa are preceded by single letter code indicating microbial domain (A = Archaea, B = Bacteria, E = Microeukarya) and two letter code indicating taxonomic depth of classification, if other than genus (sg = supergroup, do = domain, ph = phylum, cl = class, or = order, fa = family).

^c^Cluster association in network Fig. 3.

**Fig. 4:**
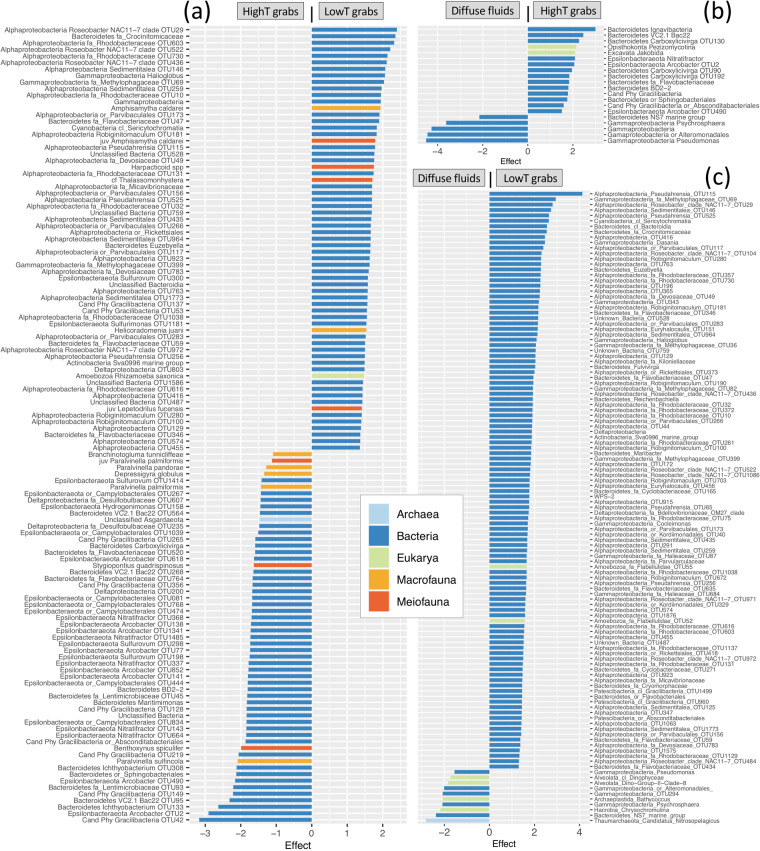
Bacteria, archaea, microeukarya, macrofauna and meiofauna responsible for significant differences between sample types. **a** High temperature (>25 °C) versus low temperature (<25 °C) grab samples, **b** high temperature grab samples versus associated diffuse fluids, and **c** low temperature grab samples versus associated diffuse fluids. Effect size differences indicate relative enrichment in one sample type over the other. Only effect size differences ≥1 are shown. OTUs enriched in diffuse fluids include only those that were also enriched relative to background fluids.

Assignment of microbial taxa (OTUs binned by identical taxonomic assignments) to core communities was primarily based on covariance with highT- or lowT-enriched fauna. Enrichment of individual OTUs within those taxa in the same temperature regime—highT or lowT—provided additional evidence and strengthened the assignment. Numerous microbial OTUs were notably enriched in some sample types (Fig. 4, Supplementary Table 4). Due to frequent low relative abundances of archaeal and microeukaryal rRNA genes, many OTUs identified as enriched in individual domain ALDEx2 tests (see Supplementary Fig. 9) did not remain so in the combined microbial assemblage results. HighT-enriched microbes included various Epsilonbacteraeota and microeukaryotes belonging to the Excavata and Fungi. Actinobacteria, Alpha- and Gammaproteobacteria, and Amoebozoa were enriched only in lowT grabs. Microbial groups with mixed highT and lowT enrichment included Bacteroidetes, Deltaproteobacteria, and the superphylum Patescibacteria.

The microbial taxa for which there was the strongest enrichment and covariance evidence for inclusion in the highT core community were the genera *Nitratifractor* and *Arcobacter* (Epsilonbacteraeota) and *Carboxylicivirga* (Bacteroidetes), followed by Bacteroidetes belonging to the Sphingobacteriales and BD2-2 and VC2.1 Bac22 clades (Table 3). Those covarying with highT fauna but with more limited enrichment included *Hydrogenimonas* and unclassified Campylobacterales (Epsilonbacteraeota), *Maritimimonas*, *Ichthyobacterium*, and Lentimicrobiaceae (Bacteroidetes), Desulfobulbaceae (Deltaproteobacteria), unclassified Asgard group Archaea, jakobid nanoflagellates, and fungi in the order Pezizomycotina. Moderate association with highT samples (covariance only) occurred for various members of the Archaea, Aquificae, Bacteroidetes, Calditrichaeota, Deltaproteobacteria, Epsilonbacteraeota, Thermodesulfobacteria, Amoebozoa and Fungi (Table 3).

Enrichment and covariance results most strongly supported the placement of Sva0996 marine group Actinobacteria and *Halioglobus* in the lowT core community, followed by *Euzebyella* (Bacteroidetes), the family Devosiaceae (Alphaproteobacteria), a basal cyanobacteria, and, with limited enrichment, *Dasania* (Gammaproteobacteria). Microbial LowT community members with moderate associations included members of the Actinobacteria, Delta- and Gammaproteobacteria, the candidate phylum Peregrinibacteria, ciliates and cercozoa (Table 3).

### Fluid and tubeworm-hosted microbial assemblages

**Fig. 5:**
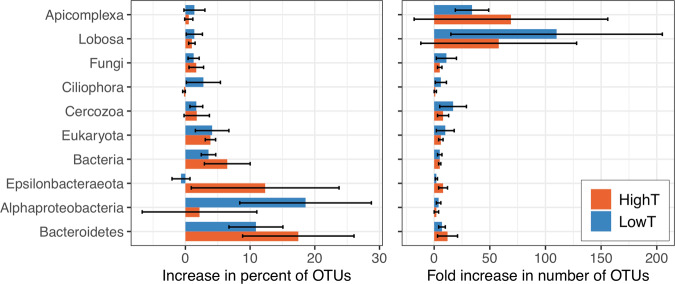
Increased diversity of major groups of Bacteria and Eukarya in highT and lowT tubeworm grabs relative to diffuse fluids. Increases shown as (**a**) the average change in the percentage of total OTUs per sample, and (**b**) the average fold change in the numbers of OTUs per sample. Error bars indicate one standard deviation.

Comparisons of the seven diffuse fluid and associated tubeworm grab samples revealed elevated microbial OTU richness in the tubeworm grabs consistently for bacteria and occasionally for microeukaryotes. Archaeal richness in the grabs was rarely elevated relative to fluids. The greatest increases in the relative proportion of OTUs per sample from fluids to tubeworm grabs occurred in the taxa shown in Fig. 5a. Collectively, these ten groups increased from 26(±5)% (fluid) to 72(±6)% (tubeworm grab) of the total OTUs per sample. Although these increases were greatest for the Bacteroidetes and Alphaproteobacteria, much smaller percentage increases for the apicomplexans and lobose amoeba amounted to nearly 50- and 90-fold changes, respectively, in the numbers of OTUs per sample (Fig. 5b). For details of the major contributors to richness increases, see Supplementary Fig. 10.

Microbial taxa that were absent from diffuse and background fluids but present in at least three of these seven associated tubeworm grabs included various Amoebozoa (10 OTUs), the candidate bacterial phyla Eremiobacteraeota (17 OTUs) and Hydrogenedentes (1 OTU), Hilomonadea (Apusozoa; 6 OTUs), and the class Breviatea (68 OTUs). Many were low abundance OTUs with limited distributions, but some within *Ca*. Eremiobacteraeota and Breviatea were widely distributed across our grab samples. *Ca*. Eremiobacteraeota OTUs occurred across lowT and one highT samples (EMw1), whereas Breviatea OTUs only occurred in grab samples with basal temperatures above 15 °C.

## Discussion

Our coordinated analysis of a broad size spectrum of organisms associated with the foundation species *Ridgeia piscesae* has defined a robust temperature-driven distinction of communities. While faunal compositional differences are known among *R. piscesae* assemblages [39, 64], our study greatly extends the characterization. Differences between assemblages with basal temperatures above and below ~25 °C were evident in all three size classes comprising characteristic highT and lowT fauna and microbes. The thermal regime is likely one of several covarying controlling factors as the chemical milieu of the habitat also affects resident species. For example, temperature is a proxy for sulphide concentration [65], which is beneficial to sulphide-oxidizing primary producers, including faunal symbionts, but may inhibit species sensitive to its toxic effects. Similarly, dissolved oxygen concentrations at vents, inversely related to temperature [66], are another driver of species distribution. Thus, we recognize that the highT/lowT designation may reflect geochemical differences not addressed in this study.

Detailed structural evaluation of these faunal assemblages was a necessary first step towards understanding interactions among species and eventual ecosystem characterization. We discuss assemblage structure, including consideration of the fluid-associated microbial context in which they reside, and then make functional inferences based on identified core communities that can be explored in more detail in the future using metagenomic and other approaches.

### Structural characterization

Taxonomic definition of the core communities associated with two primary habitat states—highT and lowT—involved distinguishing microbial residents of assemblages from those both in diffuse fluids venting through them and in background fluids. Elevated microbial OTU richness within tubeworm matrices relative to hydrothermal fluids discharging through them (Fig. 5) indicates a facilitative effect of faunal assemblages on microbial diversity. Faunal assemblages contribute to habitat stability, some remaining nearly unchanged for at least a decade [67]. At Endeavour, one estimated age of the tubeworms supporting a low temperature assemblage was 30 years [68]. We hypothesize that these relatively stable tubeworm habitats aggregate microbial diversity over years to decades from an ever-changing supply of microbial taxa in discharging hydrothermal fluids [18, 62, 69]. This aggregating effect broadens the importance of tubeworm habitats to the biodiversity of hydrothermal ecosystems from prior work on faunal diversity [37].

Furthermore, we identify tubeworm assemblages as hotspots of microbial novelty. Relative to fluids, tubeworm assemblages had increased richness of OTUs with shallow classification (domain, phylum, subphylum, class; Supplementary Fig. 10). Large numbers of OTUs classifiable only as Bacteria or Eukarya occurred exclusively in tubeworm-hosted assemblages. Richness enhancements in unclassified Alphaproteobacteria, Bacteroidia, Pezizomycotina, Apicomplexa, Cercozoa and Spirotrichea, highlight the degree to which such hydrothermal vent habitats have been under-sampled. These highly novel taxa represent unknown ecosystem contributions, interspecies interactions, and potential benefits beyond the hydrothermal ecosystem through undiscovered genetic resource potential [70]. This increased microbial richness in *R. piscesae*-hosted assemblages, with the substantial included novelty, suggests that previous assessments of hydrothermal vent microbiomes [44] may have missed the largest reservoir of diversity by not including contributions from non-symbiotic microbes in faunal assemblages.

Although we identify two primary habitat states, there is evidence of sub-components within the highT core community, suggesting even more specialized structuring and interaction of microbes and metazoans, under severe hydrothermal conditions. We propose that such structuring reflects core community members inhabiting different environmental niches within highT tubeworm grabs. Two highT sub-clusters (Fig. 3) contrasted in faunal relative abundance distributions across the sampled range, with highT-specialist fauna in cluster 1 and broader-range fauna in cluster 2 (Supplementary Fig. 8). We also noted non-overlapping covariance patterns between extreme-tolerant fauna and microbes. The copepod *Benthoxynus spiculifer* has a high oxygen affinity haemoglobin adaptation [71] to severely hypoxic conditions [72] and covaries with obligate and facultatively anaerobic (hyper)thermophiles (e.g., *Nitratiruptor*, *Thermococcus, Methanocaldococcus*) and acidophiles (e.g., Aciduliprofundaceae, *Nitratifractor, Hydrogenimonas*), bespeaking extreme (high temperature, low pH, oxygen-starved) habitat conditions. In contrast, the polychaete *Paralvinella sulfincola*, which is the most thermally tolerant aquatic metazoan on record [73], covaried with more mesophilic and facultatively anaerobic microbes indicating potential interactions in less extreme portions of its tolerance range.

Microbial taxa with limited enrichment—in highT or lowT grabs relative either to each other or to diffuse fluids—may indicate either a subsurface origin or general preference for any type of tubeworm habitat. Putative subsurface microbes—those enriched in highT versus lowT tubeworm grabs but also relatively abundant in diffuse fluids—included unclassified Asgard Archaea, members of the Bacteroidetes and Epsilonbacteraeota and a family of Deltaproteobacteria. A similar enrichment pattern did not occur in lowT grabs, suggesting decreased subsurface influence on microbial composition. Microbes broadly enriched in grab samples relative to diffuse fluids included jakobids and fungal Pezizomycotina (microeukaryotes) and *Dasania* (Gammaproteobacteria). These microeukaryotes are potential vent endemics [74, 75] for which we specify tubeworm assemblages as a likely habitat. *Dasania* and other Cellvibrionales are mesophilic, heterotrophic, obligate aerobes likely belonging to the heterotrophic “belt” surrounding diffuse vents [76]. Mixed affinity for highT and lowT assemblages in taxa belonging to Bacteroidetes, Deltaproteobacteria, and Patescibacteria may indicate generally important roles played by species occupying different environmental or metabolic niches.

### Inferred functional interactions among core community taxa

Refining the full taxonomic composition down to core communities offers opportunities for inferring functional relationships among enriched and covarying species. Increased congruence among size classes and decreased diversity of meiofauna, bacteria, and microeukaryotes in highT grabs suggest specialization of a few taxa, either by environmental selection or reliance on interspecies interactions. We identify eight faunal species (see Fig. 3) and microbial taxa primarily belonging to the Epsilonbacteraeota and Bacteroidetes as the core community favoring highT habitats. Some taxa within this core community are known to form functional associations in other habitats or laboratory settings, providing legitimacy to our method of identifying potentially interacting taxa. For example, tight coupling of carbon and nitrogen cycling may occur between Epsilonbacteraeota and Bacteroidetes, as in hydrothermal vent biofilms [77]. Bacteroidetes may also benefit from associations with core highT faunal species *D. globulus* and *P. palmiformis*, both mucus-secreting species potentially providing fresh organic material for these heterotrophic bacteria. Amoeboflagellates in the class Breviatea were among the taxa absent from fluids but associated with tubeworms; they are deeply-branching anaerobes/microaerophiles [78] that possess mitochondria-like hydrogenosomes [79] and form mutualisms involving hydrogen transfer with *Arcobacter* and other Epsilonbacteraeota [80]. These and other potential partnerships may be explored further using metagenomic approaches focused on specific core community taxa (e.g., *Carboxylicivirga*, *Arcobacter*, *Nitratifractor*, etc.), but the prevalence of potential mutualisms among highT core taxa suggests interspecies connections may be crucial to survival in more extreme hydrothermal habitats.

Functional links among lowT core taxa can be inferred but are fewer and less apparent. The core lowT faunal community was more weighted towards meiofauna, possibly reflecting lower thermal tolerance [81] and/or suggesting potentially relevant functional connections of meiofauna to lowT assemblages [82]. The inclusion of juveniles of three species in this core community suggests that lowT assemblages may serve as nursery areas for macrofauna. Juvenile macrofauna are included with meiofauna as they undoubtedly have different functional interactions within the community than their adult forms. For example, juvenile *L. fucensis* were very abundant in low T while the adults occur over the full range of habitats where they develop a symbiosis with a γ-proteobacterium, the extent of which depends on fluid vigour [83]. Recent interest in congruence among macrofauna, meiofauna, and microbes highlights the undervalued meiofaunal contributions to ecosystem processes that link the micro and macro worlds [84, 85]. Examples of inferred links involving meiofauna in the lowT core community include nematodes fuelling strictly aerobic alpha- and gammaproteobacterial heterotrophs through secretion of organic-rich mucus [86], and hypotrich ciliates, which covaried with several meiofaunal species, possibly acting as a preferred prey item. These and other potential meiofaunal interactions have been largely overlooked in hydrothermal habitats and may require creative experimental approaches to confirm [81, 87].

Ecosystem characterization aims to identify the key players and define their contributions to maintaining ecosystem function. Characterization of microbial, meio- and macrofaunal diversity across a range of habitats of the foundation species *Ridgeia piscesae* has revealed core highT and lowT communities with inferred interactions between species and highlighted the importance of faunal assemblages as hotspots of microbial richness in hydrothermal ecosystems. We propose definition of core communities as an important first step in moving from structural descriptions of biological assemblages to informed consideration of relevant functional interactions within complex and diverse ecosystems. Identification of core communities has also suggested potential functional contributions of small organisms, including meiofauna, which have been undervalued in deep-ocean monitoring and conservation strategies [88]. We foresee additional applicability of the core communities in vulnerability assessments for hydrothermal vent ecosystems in relation to future stressors such as seabed mining [89]. This work exposes the rich complexity and microbial underpinning of hydrothermal vent ecosystems to managers who, while familiar with phytoplankton-zooplankton-fish connections in the water column, may have a less established framework for extending inclusive, ecosystem-based approaches to vents and other deep-sea ecosystems.

## Supplementary Information

Supplementary Information

## Data Availability

All 16S/18S rRNA sequence data have been deposited in the NCBI Sequence Read Archive under project number PRJNA665742. Many faunal identifications were complemented with COI and/or 18S sequences (GenBank accession numbers MZ197534-MZ197773). Data and R code are available at http://github.com/smurdock-UVic/Emergent-communities.
