# Supplementary material for: Emergent “core communities” of microbes, meiofauna and macrofauna at hydrothermal vents
Source: ISME Commun. 2021 Jun 21;1:27. doi: 10.1038/s43705-021-00031-1 (PMC9723782; doi:10.1038/s43705-021-00031-1)
Supplement: Supplementary file 1 — Supplementary Information [file 43705_2021_31_MOESM1_ESM.pdf]

Emergent 'core communities' of microbes, meiofauna and macrofauna at hydrothermal vents

Murdock SA[1], Tunnicliffe V[1,2], Boschen-Rose RE[2,3], Juniper SK[1,2,4]

[1] School of Earth & Ocean Sciences, University of Victoria, Victoria, Canada. [2] Department of Biology, University of Victoria, Victoria, Canada. [3] Ocean & Earth Science, University of Southampton, Southampton, UK. [4] Ocean Networks Canada, University of Victoria, Victoria, Canada

Supplementary Materials and Methods

Sample collection

Locations were visually assessed to determine the likelihood of retrieving a tubeworm bush with associated fauna intact. Hydrothermal fluids venting through assemblages were collected prior to assemblage sampling using the ROPOS suction sampler with the intake positioned as close as possible to the faunal assemblage without disturbing it. Afterwards, the manipulator claw gently encompassed part, or all, of the tubeworm bush (at the base) and transferred tubes and associated organisms to a collection box which was then sealed. The entire sample was usually retrieved in a single grab. We reviewed video imagery of the operation to determine losses of mobile fauna, and subsequently added small numbers of scaleworms and a few squat lobsters to final counts.

Shipboard, approximately half of each tubeworm grab sample was placed in a bucket of cold artificial seawater (ASW) and held at 4˚C for processing of the microbial component. The microbial fraction was a mix of loosely associated cells and firmly attached biofilms that had to be detached prior to collection on filters. The collection boxes on the ROV were then drained to ensure all remaining organisms were retained, and samples were preserved in either 75% ethanol or 7% buffered formalin. See *Supplementary Figure 2* for visual representation of processing steps.

Macro- and meiofaunal characterization

To sort the material, we first assessed sample size and, in four cases, split the samples (using only ¼ to ½) to speed processing. In all these splits, species discovery rapidly reached asymptote (Supplementary Figure 4), thus it is likely we missed few or none. Next, we removed large *Ridgeia pisceae* tubes and processed the remaining material into two size classes on 1mm and 64μm sieves into fresh 75% ethanol. Macrofauna was retained on the larger sieve, while the animals on the 64μm sieve constituted the meiofauna. Examination of material passing the 64μm sieve never revealed animals. Where the adult stages of species occurred on both sieves, all individuals were assigned to the fraction that retained the most numbers. Many juveniles of macrofauna passed the 1mm sieve and are reported separately in the meiofaunal list. As meiofaunal individuals were abundant, we used a plankton splitter to generate smaller portions. For six of the nine samples, 0.25 portions were fixed for counting, but for the remaining samples, finer 0.05 to 0.10 splits were required because of high copepod abundances.

Animals were identified to distinct species for all macrofauna although complete names are not available for all. Among meiofauna, where copepods were highly abundant, subsamples of 200-250 individuals were identified to species. However, insufficient taxonomic information hampered differentiation of harpacticoid taxa, especially as juvenile forms were present. Nematodes were

separated into three morphotypes representing at least three species. We calculated relative abundances of species/taxa within each size class for every sample.

To calculate density, tube surface area was determined from images of each tubeworm following the method of Tsurumi and Tunnicliffe (2003). For similarity and cluster analyses, juveniles of macrofauna remained in the meiofauna as we are interested in the functional relations among the five groups in this study. Rarefaction curves were produced in PAST v.3.23 (Hammer et al 2001).

Microbial DNA extraction and sequencing

Primers used for paired-end sequencing on Illumina MiSeq included 63F (5'-CAGGCCTAACACATGCAAGTC-3') and 519R (5'-GWATTACCGCGGCKGCTG-3') for Bacteria, 956F (TYAATYGGANTCAACRCC) and 1410R (5'-CRGTGWGTRCAAGGRGCA-3') for Archaea, and 572F (5'-CYGCGGTAATTCCAGCTC-3') and 1009R (5'-AYGGTATCTRATCRTCTTYG-3') for Eukarya. Bacteria and Archaea were sequenced from genomic DNA but initial sequencing of Eukarya from genomic DNA returned mostly metazoan sequences. Therefore, sequence data for microeukaryotes reported in this study came from amplicons generated using the primers 18S-EUK581-F (5'-GTGCCAGCAGCCGCG-3') and 18S-EUK1134-R (5'-TTTAAGTTTCAGCCTTGCG-3') (Bower et al 2004), which have been validated for selectively amplifying non-metazoan DNA (Del Campo et al 2019). Microeukaryote 18S amplicons were produced on an iCycler (Bio-Rad) with the following conditions: initial denaturation at 94°C for 2 minutes, 27 cycles of 94°C for 30 seconds, 62°C for 45 seconds, and 72°C for 1 minute, and final extension at 72°C for 10 minutes. Each 20µl amplification reaction consisted of 1µl of DNA, 1X buffer (2.5mM $MgCl_2$; Promega), 0.2mmol each deoxyribonucleoside triphosphate (dNTP), 0.25µmol each primer, 1U of GoTaq polymerase (Promega), and DNAse-free water to final volume. Amplicons were cleaned using a QiaQuick PCR purification kit (Qiagen) prior to preparation for sequencing.

Quantitative PCR

Relative abundances of bacteria, archaea and microeukarya in each sample were determined by quantitative PCR (qPCR) of 16S and 18S rRNA genes using established and tested lab protocols, which utilize the primers 331F (5'-TCCTACGGGAGGCAGCAGT-3') and 797R (5'-GGACTACCAGGGTATCTAATCCTGTT-3') (Nadkarni et al 2002) for bacteria, 20F (5'-TTCCGGTTGATCCYGCCRG-3') and DW518R (5'-GNTTTACCGCGGCKGCTG-3') (Zaikova et al 2010) for archaea, and 18S-EUK581-F and 18S-EUK1134-R (Bower et al 2004) for microeukaryotes. To create qPCR standards, plasmid DNA was extracted from 4 bacterial, 5 archaeal, and 3 microeukaryote environmental and culture clones from a variety of hydrothermal vent locations (in-house collections from Mariana Arc, Kermadec Arc and Juan de Fuca Ridge) using a Miniprep plasmid extraction kit (Qiagen) and further prepared as described in (Zaikova et al 2010) beginning with DNase treatment. Plasmids were quantified on a Qubit 3 (Invitrogen) using the Qubit dsDNA high-sensitivity assay (Invitrogen) and mixed in equal volumes to produce a standard for each domain, with concentrations on the order of 10^7 molecules/µl.

A ten-fold dilution series of each plasmid mixture, ranging from 10^7 to 10^1, was used to produce standard curves with R^2 values > 0.995. Each 10µl reaction consisted of 5µl SsoFast EvaGreen Supermix (Bio-Rad), 0.5µM each forward and reverse primers, 2µl DNase-free water, and 1µl of DNA template. Reactions were performed in triplicate on a CFX96 Real-Time PCR Detection System (Bio-Rad) and consisted of an initial denature for 2 min at 95°C followed by 45 cycles of 95°C for 5 sec and a 5 sec anneal/extend step and concluding with a melt curve from 65-95°C. Temperatures for the anneal/extend step were 61°C for bacteria/archaea and 62°C for eukarya. Each run included a standard curve and a no-template control. Samples were analyzed with DNA combined from the two size fractions. A dilution series of each DNA sample was analyzed to test for inhibitory

reaction effects, and final quantitative reactions were run using a 1:10 dilution of the DNA. CFX Manager 2.0 software (Bio-Rad) was used to analyze the results.

Microbial dissimilarity linked to sampling method

To address whether microbial composition was influenced by different techniques used for harvesting cells from tubeworm grab samples (direct removal versus mild agitation in ASW), we performed Analysis of Similarities (ANOSIM) on two samples (EMw1 and EMw6) with replicates that were subjected to both removal methods. ANOSIM was also used to test for compositional differences between the two size fractions (0.2-20μm, 20-64μm) collected by serial filtration of microbial biomass.

Supplementary Results

Tests of sampling bias on microbial composition

Composition of the multi-domain balanced microbial assemblage was not significantly different (ANOSIM R=0.25, p=0.33) between the two harvesting methods, although when domains were tested separately using ALDEx2, a few bacterial and microeukaryal OTUs were differentially represented between the two methods (not shown). Further ANOSIM performed on all tubeworm-associated extracts found no OTUs that were consistently enriched using one method versus the other. However, removal method did have an effect on microeukaryote diversity, with significantly lower inverse Simpson values when using the direct removal method (Mann-Whitney test, p=0.004).

Serial filtration onto 20 and 0.2 μm filters was aimed at capturing eukaryotes from both micro (20-64μm) and pico/nano (0.2-20μm) size ranges, but our results indicated no significant difference (ANOSIM R=0.02, p=0.23) in composition between size fractions for eukaryotes or bacterial and archaea.

Faunal Character

The projected (assuming full sample sort) ratio of total meiofauna to macrofauna was 3.7, however, the variation among samples was very high (0.6 to 317). Overall, we identified 58 distinct taxa (*Supplementary Tables 1 & 2*), of which the harpacticoid copepods, and possibly the nemerteans, were lumped species. Rarefaction curves (*Supplementary Figure 7*) show rapid saturation in high temperature samples indicating that all species were likely recovered; the two lowest temperature samples with highest species numbers (30 to 35) have lower estimates of full species recovery.

Meiofaunal description

Meiofaunal density (not including juvenile macrofauna) on *Ridgeia* tubes ranged from 3.2 to 68.6 individuals/10cm^2 of tube surface in the Endeavour samples. Dirivultid copepods comprised about 80% of the meiofauna with *Stygiopontius quadrispinosus* dominating highT samples while harpacticoid copepods occurred mostly in lowT samples (Figure 2b). Only lowT samples returned nematodes (Chromadorea) and nemerteans (Palaeonemertea) at 6% abundance overall.

Macrofaunal description

Macrofaunal densities ranged from 0.2 to 1.8 individuals/10cm^2 of tube surface in the Endeavour samples. We retrieved 41 macrofaunal species in which abundances were heavily skewed with 90% of total counts represented by three species: two gastropods (*Lepetodrilus fucensis, Depressigyra globulus*) and one polychaete (*Paralvinella palmiformis*) (*Supplementary Table 1*). The limpet,

Lepetodrilus fucensis was the only species to occur in every sample. Relative abundances were highly variable at lowT (Figure 2c).

Supplementary References

Bower SM, Carnegie RB, Goh B, Jones SR, Lowe GJ, Mak MW (2004). Preferential PCR amplification of parasitic protistan small subunit rDNA from metazoan tissues. *J Eukaryot Microbiol* **51:** 325-332.

Del Campo J, Pons MJ, Herranz M, Wakeman KC, Del Valle J, Vermeij MJA *et al* (2019). Validation of a universal set of primers to study animal-associated microeukaryotic communities. *Environ Microbiol* **21:** 3855-3861.

Hammer Ø, Harper DAT, Ryan PD (2001). PAST: Paleontological statistics software package for education and data analysis. *Palaeontologia Electronica* **4:** 1-9.

Nadkarni MA, Martin FE, Jacques NA, Hunter N (2002). Determination of bacterial load by real-time PCR using a broad-range (universal) probe and primers set. *Microbiology* **148:** 257-266.

Tsurumi M, Tunnicliffe V (2003). Tubeworm-associated communities at hydrothermal vents on the Juan de Fuca Ridge, northeast Pacific. *Deep-Sea Research Part I-Oceanographic Research Papers* **50:** 611-629.

Zaikova E, Walsh DA, Stilwell CP, Mohn WW, Tortell PD, Hallam SJ (2010). Microbial community dynamics in a seasonally anoxic fjord: Saanich Inlet, British Columbia. *Environ Microbiol* **12:** 172-191.

Supplementary Figures

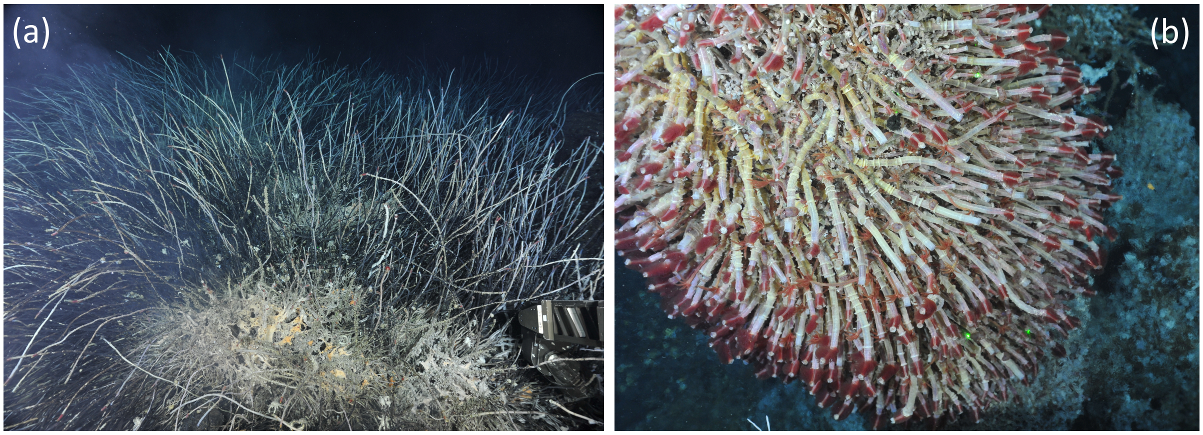

Supplementary Figure 1. Microbial and faunal assemblages hosted by the Siboglinid tubeworm *Ridgeia piscesae* in (a) low temperature, basalt-hosted and (b) high temperature, sulfide-hosted habitats.

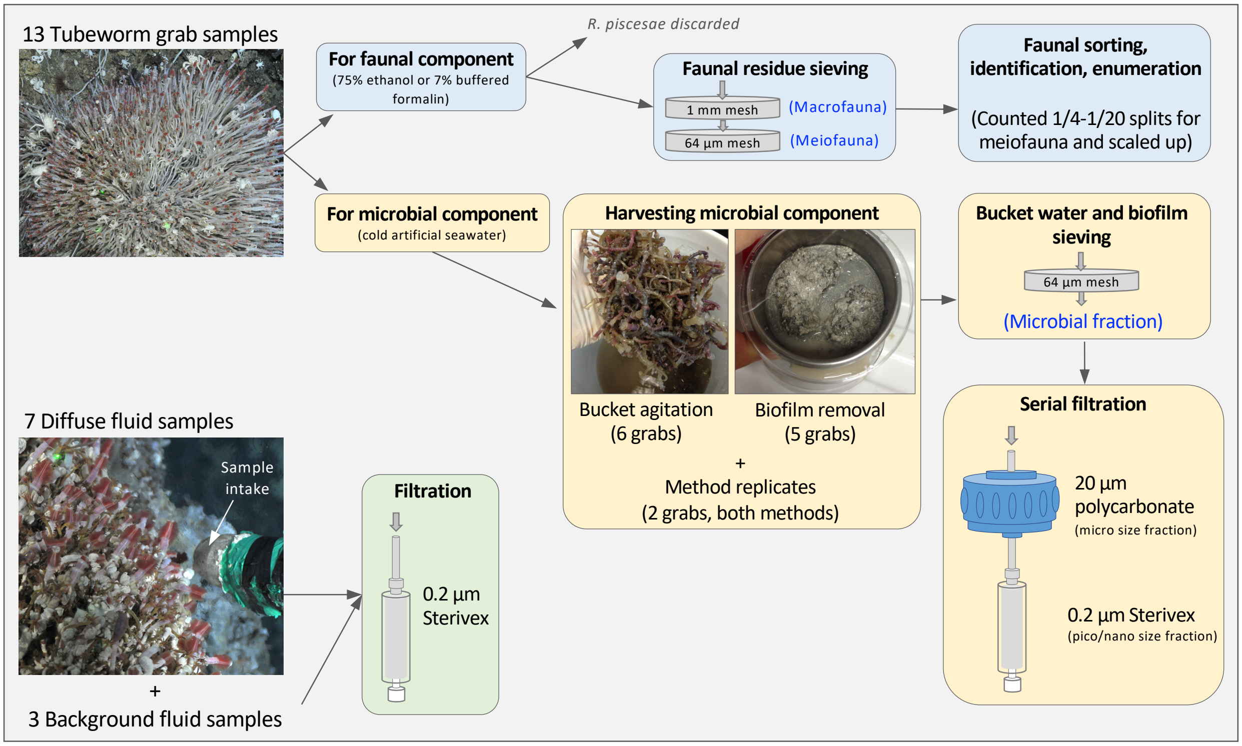

Supplementary Figure 2. Sample processing steps from tubeworm grabs and diffuse and background fluids. Tubeworm grabs were divided for processing of fauna (blue boxes) and microbes (yellow boxes), and fluid samples went straight to filtration (green box).

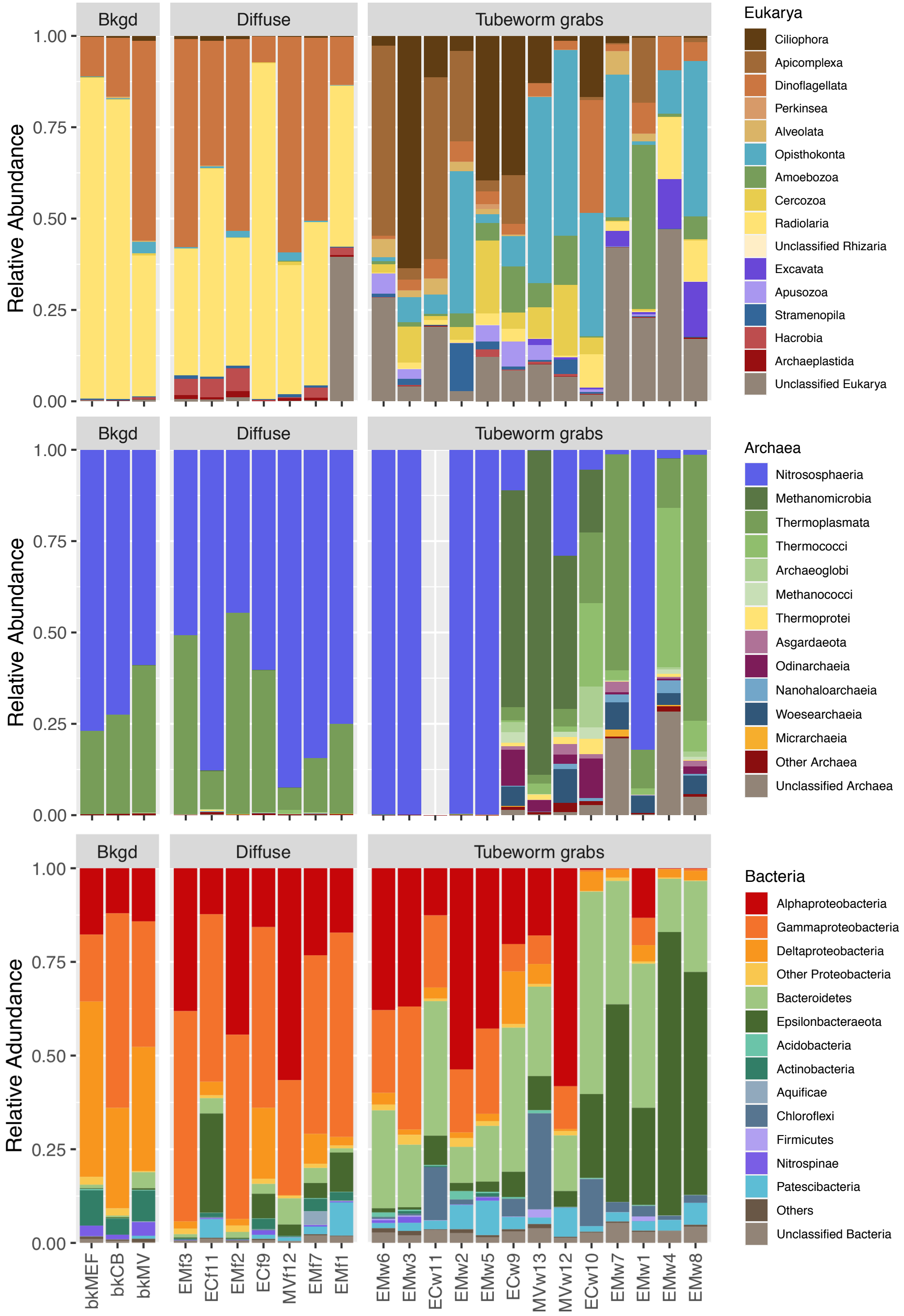

Supplementary Figure 3. Taxonomic identity and relative abundance of sequences for each domain. Sample ECw11 did not produce sufficient archaeal reads. Bkgd= background fluid samples; Diffuse= diffuse fluid samples; Tubeworm grabs= grab samples. Diffuse fluid and grab samples are arranged by increasing basal temperature from left to right.

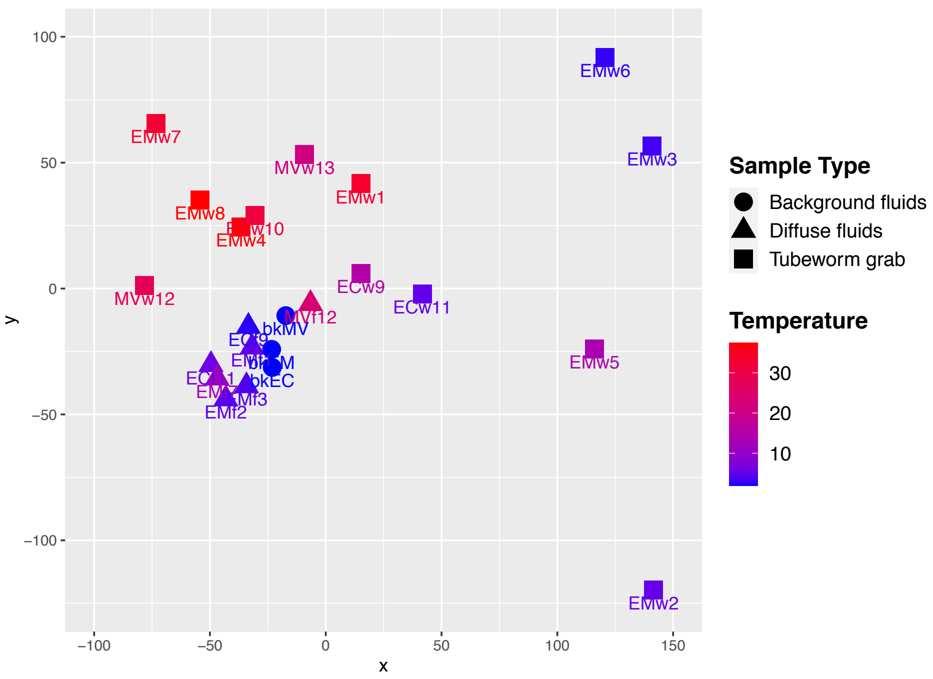

Supplementary Figure 4. Nonmetric Multidimensional Scaling ordination of the q-PCR balanced microbial assemblage from 13 grab samples, 7 diffuse fluid and 3 background fluid samples. 2-D stress=0.13.

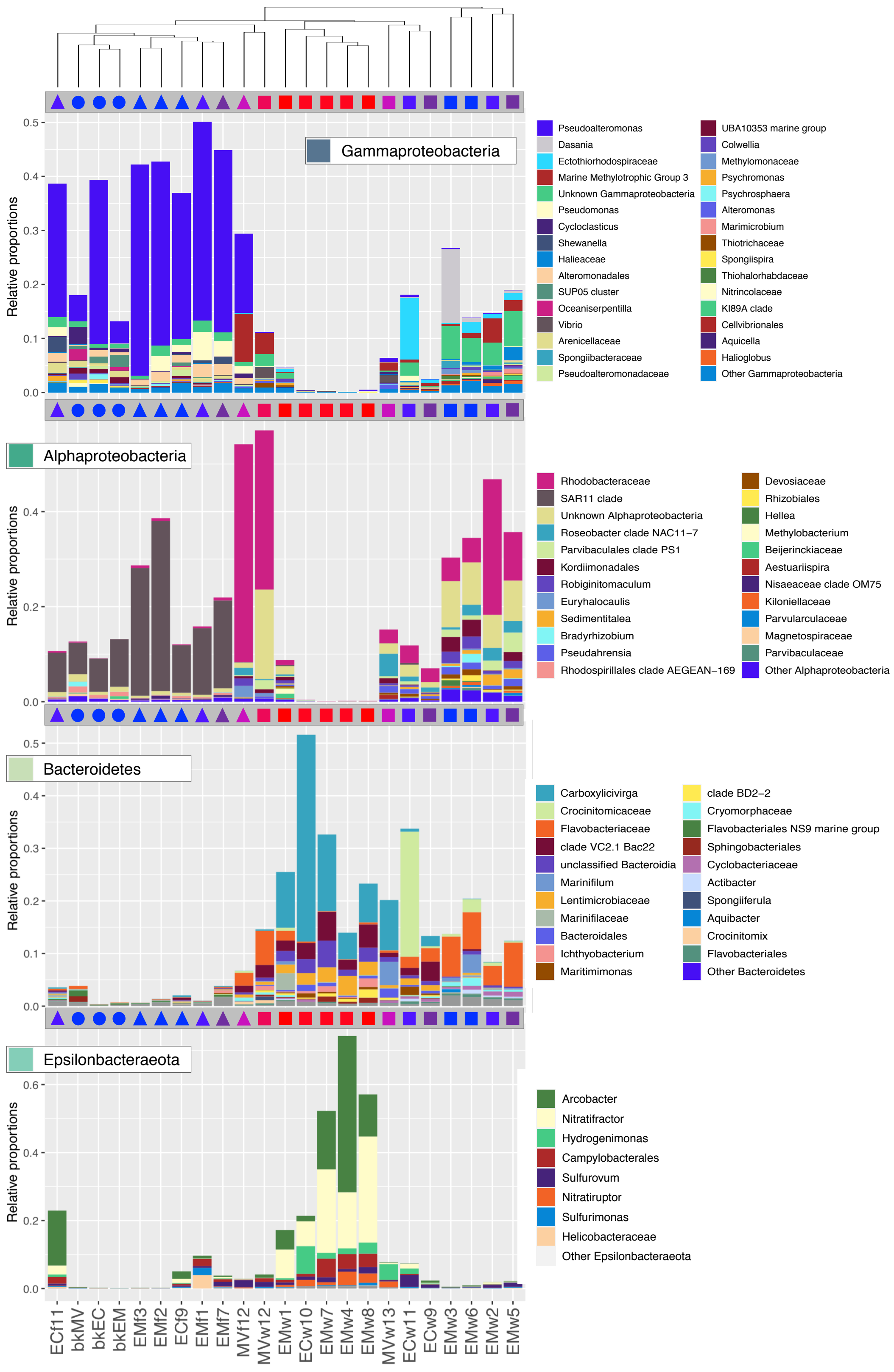

Supplementary Figure 5. Taxonomic breakdown of the four bacterial groups with the greatest contributions to diversity (from Figure 2) across the sample set. Sample order and dendrogram are identical to Figure 2a.

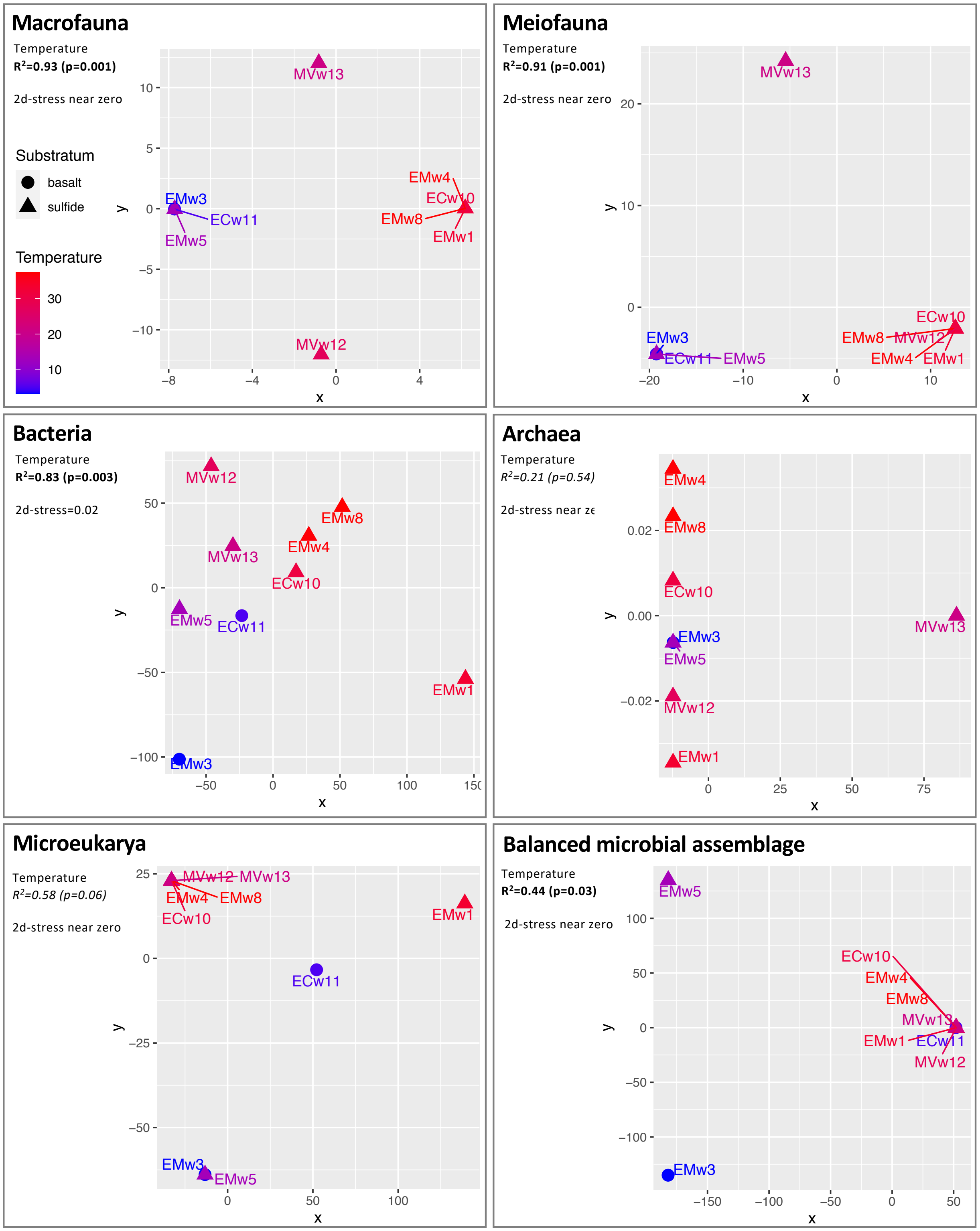

Supplementary Figure 6. Nonmetric Multidimensional Scaling ordinations used in Procrustes analysis. Ordinations were performed using Aitchison distances between samples with centered log ratio transformed abundances.

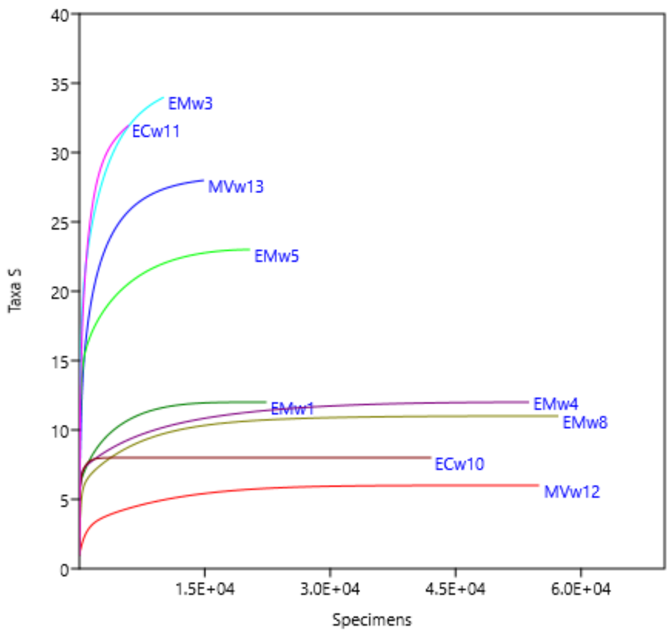

Supplementary Figure 7. Rarefaction of individuals (macro- and meiofauna) within samples; unique species (i.e., juveniles not included) only. Five highT samples reach saturation rapidly, two more lowT (sulfide) slowly and two lowT (basalt) not at all.

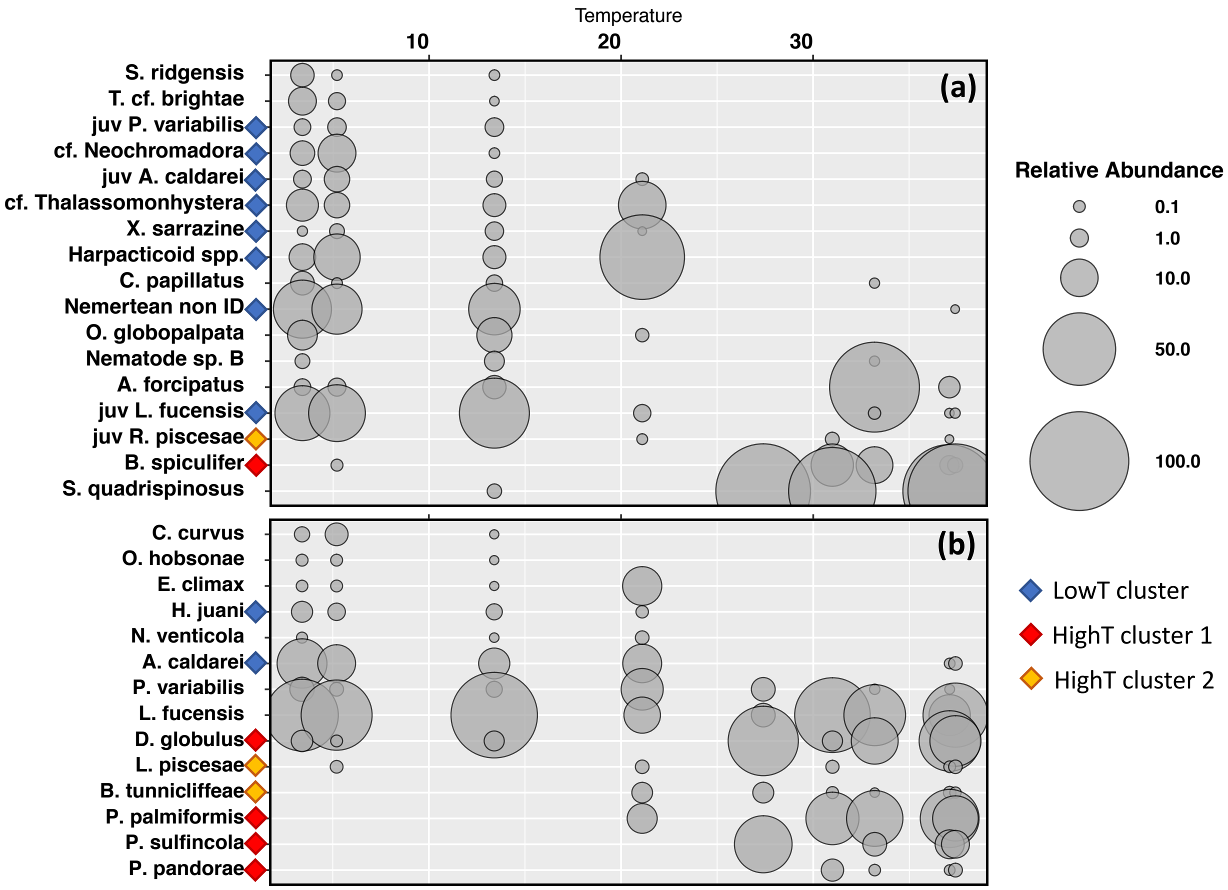

Supplementary Figure 8. Relative abundance (within each size class) of common (a) meiofauna and (b) macrofauna across the temperature range of sampled *Ridgeia piscesae* assemblages. Taxa with colored diamonds belong to either the lowT or highT community based on enrichment or covariance with an enriched species. Red and orange colors indicate the highT network cluster in which the species falls in Figure 3.

Supplementary Figure 9. Enriched taxa identified and combined from ALDEx2 tests run on individual microbial domains. Bacteria, archaea, microeukarya, macro- and meiofauna responsible for significant differences between (a) highT and lowT (above and below 25°C, respectively) tubeworm grabs, (b) lowT grabs and associated diffuse fluids, (c) highT grabs and associated diffuse fluids, and (d) diffuse and background fluids. Effect size differences indicate relative enrichment in one sample type over the other. Only effect size differences ≥1 are shown.

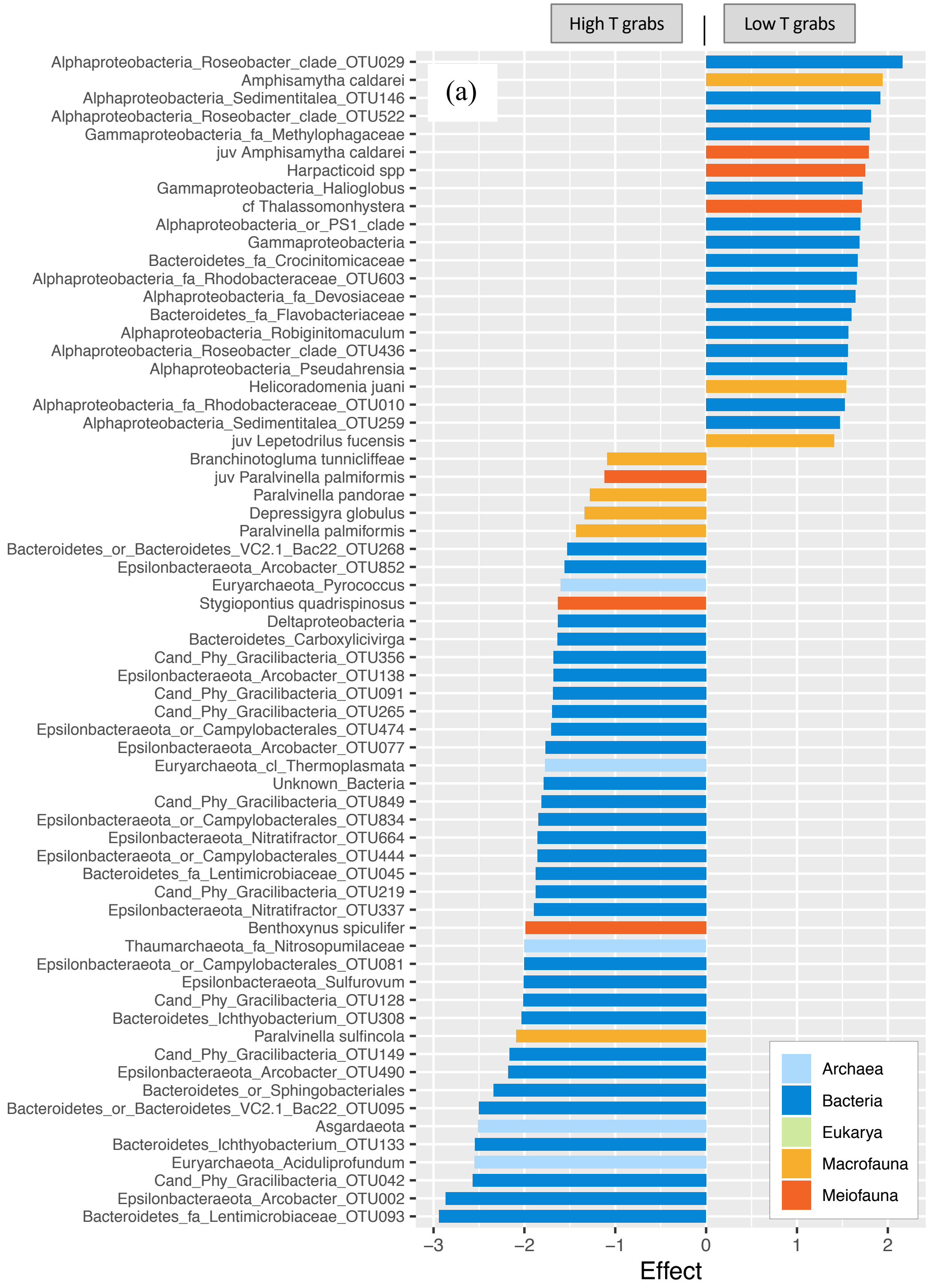

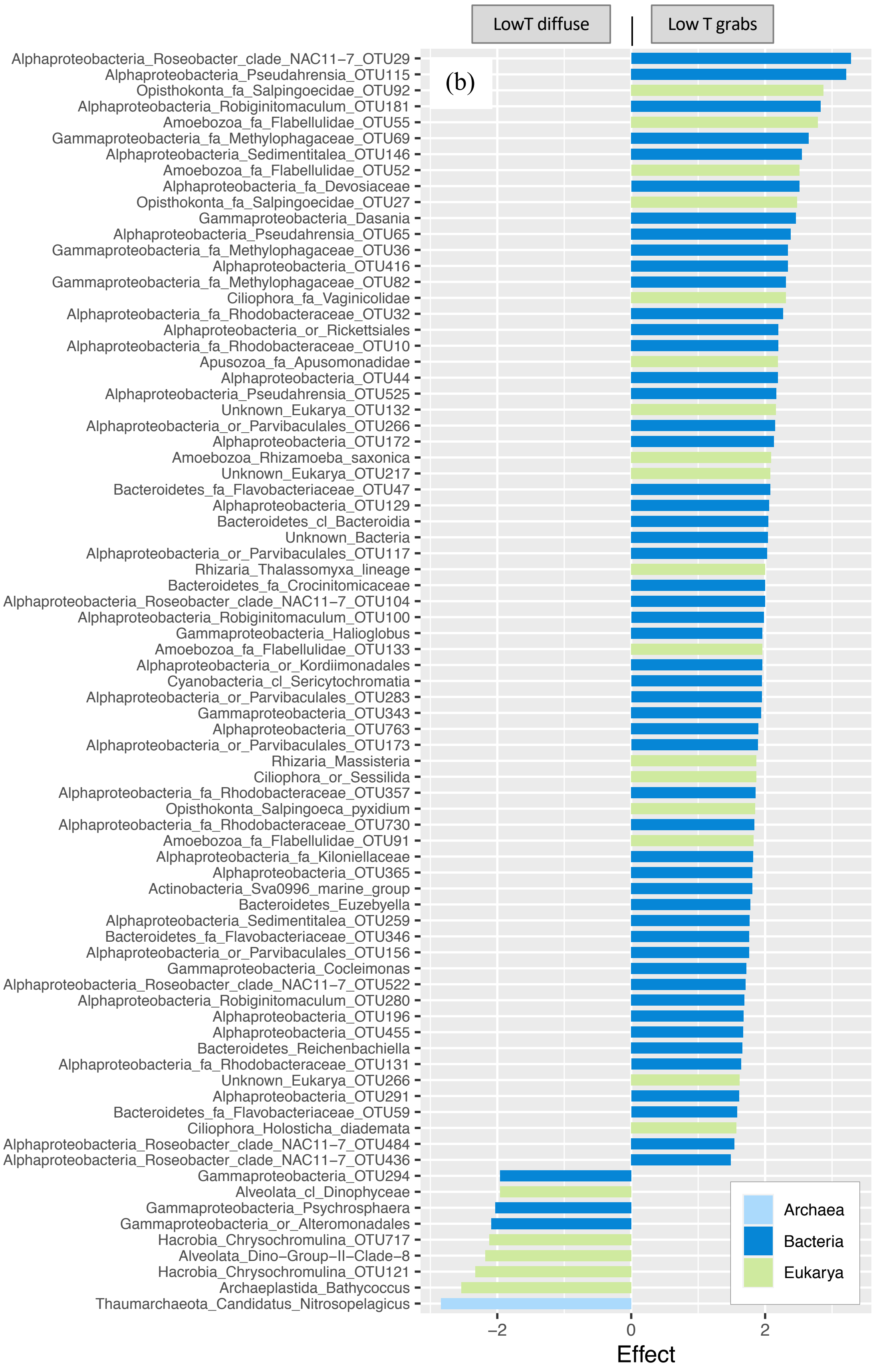

LowT diffuse
Low T grabs
(b)
Alphaproteobacteria_Roseobacter_clade_NAC11−7_OTU29
Alphaproteobacteria_Pseudahrensia_OTU115
Opisthokonta_fa_Salpingoecidae_OTU92
Alphaproteobacteria_Robiginitomaculum_OTU181
Amoebozoa_fa_Flabellulidae_OTU55
Gammaproteobacteria_fa_Methylophagaceae_OTU69
Alphaproteobacteria_Sedimentitalea_OTU146
Amoebozoa_fa_Flabellulidae_OTU52
Alphaproteobacteria_fa_Devosiaceae
Opisthokonta_fa_Salpingoecidae_OTU27
Gammaproteobacteria_Dasania
Alphaproteobacteria_Pseudahrensia_OTU65
Gammaproteobacteria_fa_Methylophagaceae_OTU36
Alphaproteobacteria_OTU416
Gammaproteobacteria_fa_Methylophagaceae_OTU82
Ciliophora_fa_Vaginicolidae
Alphaproteobacteria_fa_Rhodobacteraceae_OTU32
Alphaproteobacteria_or_Rickettsiales
Alphaproteobacteria_fa_Rhodobacteraceae_OTU10
Apusozoa_fa_Apusomonadidae
Alphaproteobacteria_OTU44
Alphaproteobacteria_Pseudahrensia_OTU525
Unknown_Eukarya_OTU132
Alphaproteobacteria_or_Parvibaculales_OTU266
Alphaproteobacteria_OTU172
Amoebozoa_Rhizamoeba_saxonica
Unknown_Eukarya_OTU217
Bacteroidetes_fa_Flavobacteriaceae_OTU47
Alphaproteobacteria_OTU129
Bacteroidetes_cl_Bacteroidia
Unknown_Bacteria
Alphaproteobacteria_or_Parvibaculales_OTU117
Rhizaria_Thalassomyxa_lineage
Bacteroidetes_fa_Crocinitomicaceae
Alphaproteobacteria_Roseobacter_clade_NAC11−7_OTU104
Alphaproteobacteria_Robiginitomaculum_OTU100
Gammaproteobacteria_Halioglobus
Amoebozoa_fa_Flabellulidae_OTU133
Alphaproteobacteria_or_Kordiimonadales
Cyanobacteria_cl_Sericytochromatia
Alphaproteobacteria_or_Parvibaculales_OTU283
Gammaproteobacteria_OTU343
Alphaproteobacteria_OTU763
Alphaproteobacteria_or_Parvibaculales_OTU173
Rhizaria_Massisteria
Ciliophora_or_Sessilida
Alphaproteobacteria_fa_Rhodobacteraceae_OTU357
Opisthokonta_Salpingoeca_pyxidium
Alphaproteobacteria_fa_Rhodobacteraceae_OTU730
Amoebozoa_fa_Flabellulidae_OTU91
Alphaproteobacteria_fa_Kiloniellaceae
Alphaproteobacteria_OTU365
Actinobacteria_Sva0996_marine_group
Bacteroidetes_Euzebyella
Alphaproteobacteria_Sedimentitalea_OTU259
Bacteroidetes_fa_Flavobacteriaceae_OTU346
Alphaproteobacteria_or_Parvibaculales_OTU156
Gammaproteobacteria_Cocleimonas
Alphaproteobacteria_Roseobacter_clade_NAC11−7_OTU522
Alphaproteobacteria_Robiginitomaculum_OTU280
Alphaproteobacteria_OTU196
Alphaproteobacteria_OTU455
Bacteroidetes_Reichenbachiella
Alphaproteobacteria_fa_Rhodobacteraceae_OTU131
Unknown_Eukarya_OTU266
Alphaproteobacteria_OTU291
Bacteroidetes_fa_Flavobacteriaceae_OTU59
Ciliophora_Holosticha_diademata
Alphaproteobacteria_Roseobacter_clade_NAC11−7_OTU484
Alphaproteobacteria_Roseobacter_clade_NAC11−7_OTU436
Gammaproteobacteria_OTU294
Alveolata_cl_Dinophyceae
Gammaproteobacteria_Psychrosphaera
Gammaproteobacteria_or_Alteromonadales
Hacrobia_Chrysochromulina_OTU717
Alveolata_Dino−Group−II−Clade−8
Hacrobia_Chrysochromulina_OTU121
Archaeplastida_Bathycoccus
Thaumarchaeota_Candidatus_Nitrosopelagicus
−2
0
2
Effect
Archaea
Bacteria
Eukarya

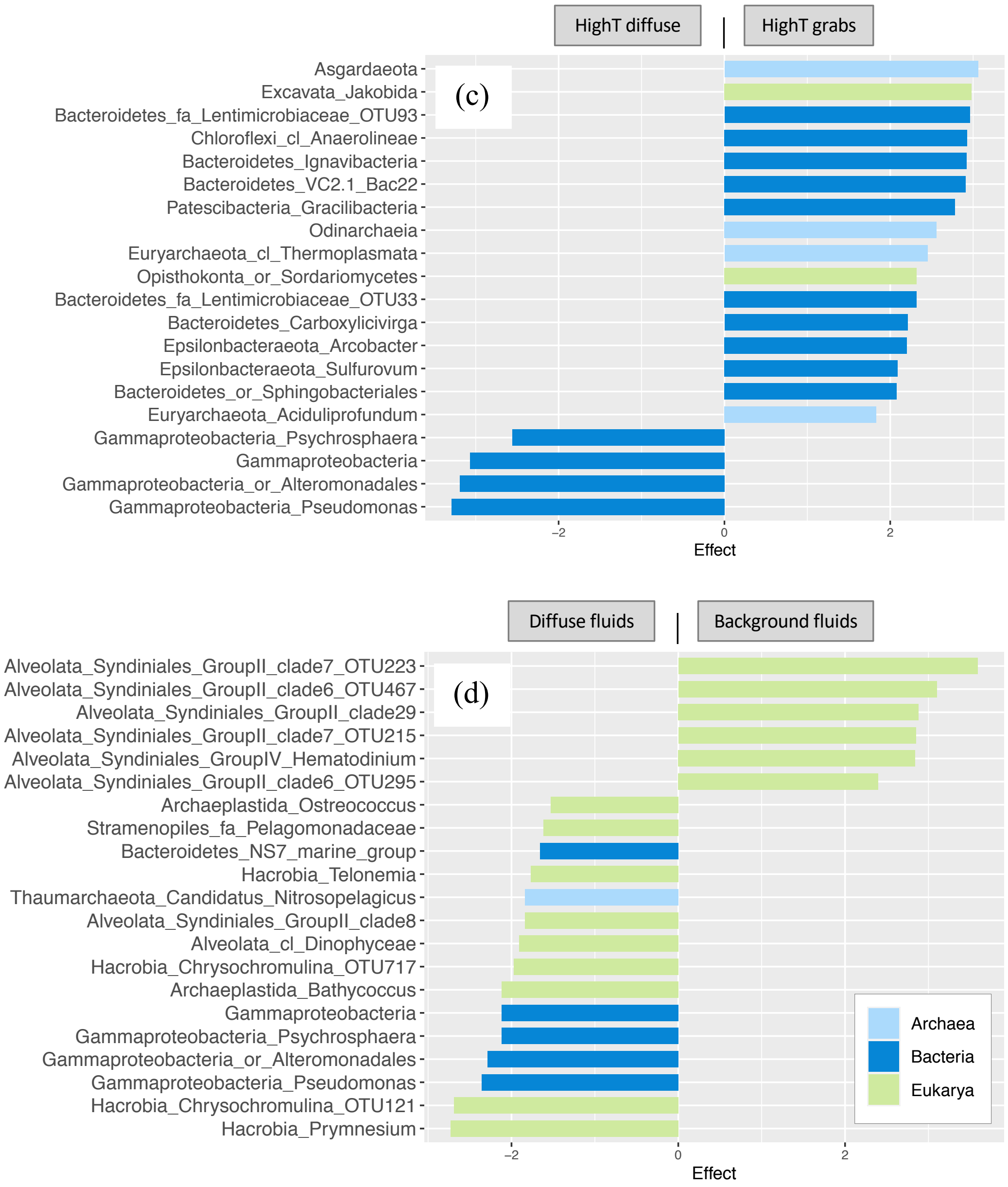

HighT diffuse
HighT grabs
(c)
Asgardaeota
Excavata_Jakobida
Bacteroidetes_fa_Lentimicrobiaceae_OTU93
Chloroflexi_cl_Anaerolineae
Bacteroidetes_Ignavibacteria
Bacteroidetes_VC2.1_Bac22
Patescibacteria_Gracilibacteria
Odinarchaeia
Euryarchaeota_cl_Thermoplasmata
Opisthokonta_or_Sordariomycetes
Bacteroidetes_fa_Lentimicrobiaceae_OTU33
Bacteroidetes_Carboxylicivirga
Epsilonbacteraeota_Arcobacter
Epsilonbacteraeota_Sulfurovum
Bacteroidetes_or_Sphingobacteriales
Euryarchaeota_Aciduliprofundum
Gammaproteobacteria_Psychrosphaera
Gammaproteobacteria
Gammaproteobacteria_or_Alteromonadales
Gammaproteobacteria_Pseudomonas
−2
0
2
Effect
Diffuse fluids
Background fluids
(d)
Alveolata_Syndiniales_GroupII_clade7_OTU223
Alveolata_Syndiniales_GroupII_clade6_OTU467
Alveolata_Syndiniales_GroupII_clade29
Alveolata_Syndiniales_GroupII_clade7_OTU215
Alveolata_Syndiniales_GroupIV_Hematodinium
Alveolata_Syndiniales_GroupII_clade6_OTU295
Archaeplastida_Ostreococcus
Stramenopiles_fa_Pelagomonadaceae
Bacteroidetes_NS7_marine_group
Hacrobia_Telonemia
Thaumarchaeota_Candidatus_Nitrosopelagicus
Alveolata_Syndiniales_GroupII_clade8
Alveolata_cl_Dinophyceae
Hacrobia_Chrysochromulina_OTU717
Archaeplastida_Bathycoccus
Gammaproteobacteria
Gammaproteobacteria_Psychrosphaera
Gammaproteobacteria_or_Alteromonadales
Gammaproteobacteria_Pseudomonas
Hacrobia_Chrysochromulina_OTU121
Hacrobia_Prymnesium
−2
0
2
Effect
Archaea
Bacteria
Eukarya

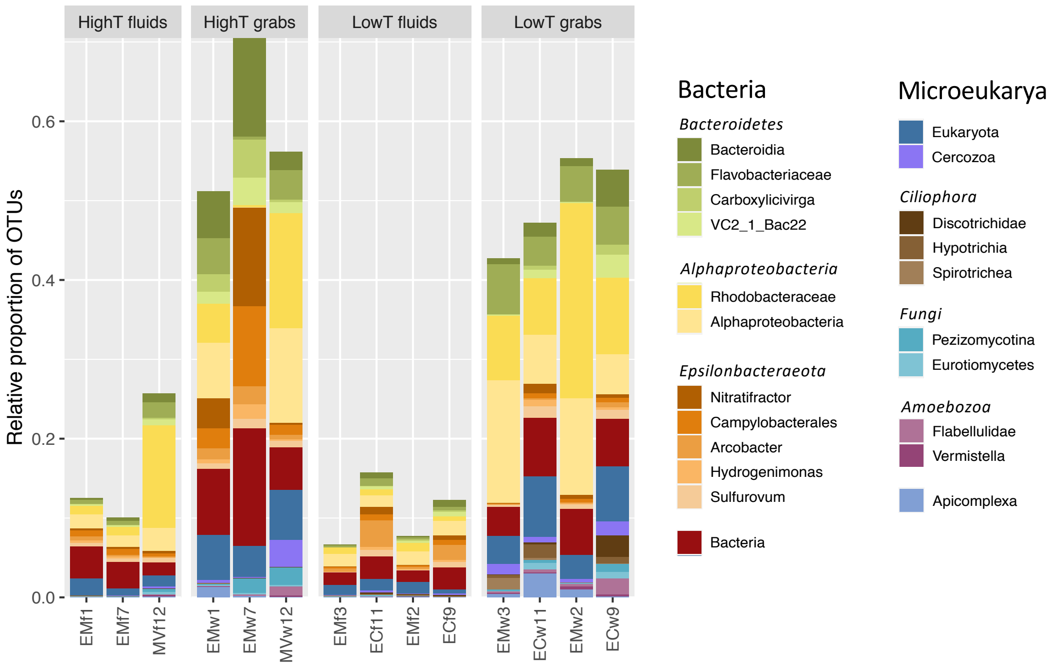

Supplementary Figure 10. Largest microbial contributors to OTU richness differences between fluid and grab samples.

Supplementary Tables

Supplementary Table 1. Macrofaunal species abundances (counts justified to full sample).

Group	Family	Name	EMw1	EMw3	EMw4	EMw5	EMw8	ECw10	ECw11	MVw12	MVw13
Amphipoda	Ischyroceridae	*Bonnieriella linearis*	0	0	0	0	0	0	1	0	0
Amphipoda	Pardaliscidae	*Pardalisca endeavouri*	0	63	0	0	0	0	52	0	0
Amphipoda	Sebidae	*Seba profunda*	0	2	0	0	0	0	6	0	0
Asteroidea	Xyloplactidae	*Xyloplax* n. sp.	0	46	0	0	0	0	0	0	0
Bivalvia	Benthomodiolinae	*Benthomodiolus erebus*	0	0	0	0	0	0	2	0	11
Bivalvia	Vesicomyidae	unk juveniles	0	0	0	0	0	0	0	0	2
Cnidaria	Actinaria	unk anemone	0	0	0	0	0	0	0	0	22
Decapoda	Munidopsidae	*Munidopsis alvisca*	0	0	0	0	0	0	2	0	103
Demospongiae	Cladorhizidae	Cladorhizidae	0	3	0	0	0	0	0	0	0
Foraminifera		Foraminifera	0	1	0	0	0	0	7	0	0
Gastropoda	Buccinidae	*Buccinum thermophilum*	0	2	0	0	0	0	0	0	0
Gastropoda	Lepetodrilidae	*Clypeosectus curvus*	0	18	0	2	0	0	63	0	0
Gastropoda	Lepetodrilidae	*Lepetodrilus fucensis*	5016	1534	716	10370	1020	17446	1169	6	140
Gastropoda	Peltospiridae	*Depressigyra globulus*	2612	65	1834	234	585	538	4	96	0
Gastropoda	Provannidae	*Provanna variabilis*	8	96	2	104	0	0	9	6	200
Gastropoda	Provannidae	*Provanna cf laevis*	0	0	0	0	0	0	0	0	15
Gastropoda	Pyropeltidae	*Pyropelta musaica*	0	0	0	0	0	0	0	0	3
Gastropoda	Skeneidae	*Fucaria striata*	0	1	0	0	0	0	5	0	117
Hydrozoa		Hydrozoa	0	8	0	0	0	0	407	0	0
Ostracoda	Myodocopida	*Euphilomedes climax*	0	4	0	2	0	0	4	0	167
Polychaeta	Allvinellidae	*Paralvinella dela*	0	0	0	0	0	0	0	0	2
Polychaeta	Allvinellidae	*Paralvinella palmiformis*	4064	0	1676	0	445	7711	0	0	83
Polychaeta	Allvinellidae	*Paralvinella pandorae*	20	0	4	0	10	758	0	0	0
Polychaeta	Allvinellidae	*Paralvinella sulfincola*	424	0	284	0	123	0	0	61	0
Polychaeta	Ampharetidae	*Amphisamytha carldarei*	0	640	4	884	9	0	260	0	164
Polychaeta	cf Archinomidae	sp non ID	0	0	0	0	0	0	0	0	4
Polychaeta	Hesionidae	sp non ID	0	0	0	0	0	0	0	0	9
Polychaeta	Maldanidae	*Nicomache venticola*	0	3	0	4	0	0	0	0	5
Polychaeta	Maldanidae	*Nicomache cf ardwissoni*	0	0	0	0	0	0	0	0	5
Polychaeta	Orbiniidae	*Orbiniella hobsonae*	0	5	0	2	0	0	4	0	0
Polychaeta	Orbiniidae	*Scoloplos* n. sp.	0	3	0	0	0	0	0	0	0
Polychaeta	Phyllodocidae	*Protomystides verenae*	0	0	0	0	0	0	21	0	112
Polychaeta	Polynoidae	*Branchinotogluma tunnicliffeae*	4	0	12	0	3	59	0	4	27
Polychaeta	Polynoidae	*Lepidonotopodium piscesae*	0	0	8	0	9	90	6	0	5
Polychaeta	Polynoidae	*Levensteiniella intermedia*	0	0	0	4	0	0	6	0	0
Polychaeta	Scolicida	sp non ID	0	0	0	0	0	0	0	0	8
Polychaeta	Spionidae	sp non ID	0	0	0	0	0	0	0	0	8
Pycnogonida	Ammotheidae	*Sericosura dissita*	0	1	0	0	0	0	0	0	0
Pycnogonida	Ammotheidae	*Sericosura verenae*	0	2	0	6	0	0	1	0	0
Sipuncula		Sipunculidea	0	5	0	0	0	0	1	0	0
Solenogastres	Simrothiellidae	*Helicoradomenia juani*	0	61	0	106	0	0	25	0	3
		**Justified to full sample**	12148	2563	4540	11718	2204	26602	2055	173	1215
		**Original numbers assessed**	3037	2563	2270	5859	2204	10641	2055	173	1215
		**# spp present**	7	21	9	11	8	6	21	5	23

Supplementary Table 2. Meiofaunal species abundances (counts justified to full sample).

Group	Family	Name	EMw1	EMw3	EMw4	EMw5	EMw8	ECw10	ECw11	MVw12	MVw13
Arachnida	Halacaridae	*Copidognathus papillatus*	4	336	0	150	0	0	4	0	0
Hexanauplia	Dirivultidae	*Aphotopontius forcipatus*	9184	96	1088	540	0	0	64	0	0
Hexanauplia	Dirivultidae	*Benthoxynus spiculifer*	1096	0	726	0	312	2408	8	0	0
Hexanauplia	Dirivultidae	*Stygiopontius quadrispinosus*	0	0	47530	84	54796	13186	0	54912	0
Hexanauplia	Dirivultidae	*Collocherides brychius*	0	200	0	0	0	0	0	0	0
Hexanauplia	Harpacticoida	spp	0	480	0	512	0	0	1036	0	10840
Malacostraca	Pardaliscidae	*Pardalisca endeavouri*	0	64	0	0	0	0	132	0	0
Malacostraca	Amphipoda	Amphipod sp (Seba?)	0	20	0	0	0	0	0	0	0
Ostracoda	Cytheruridae	*Xylocethere sarrazinae*	0	4	0	240	0	0	28	0	1
Ostracoda	Pontocyprididae	*Thomontocypris cf brightae*	0	549	0	4	0	0	52	0	0
Gastropoda	Lepetodrilidae	*Lepetodrilus fucensis*	20	3184	12	9456	20	0	1688	0	140
Gastropoda	Peltospiridae	*Depressigyra globulus*	0	0	0	4	0	0	16	0	0
Gastropoda	Provannidae	*Provanna variabilis*	0	96	0	252	0	0	72	0	0
Polychaeta	Alvinellidae	*Paralvinella palmiformis*	0	0	8	0	0	98	0	0	0
Polychaeta	Ampharetidae	*Amphisamytha carldarei*	0	124	0	140	0	0	204	0	30
Polychaeta	Dorvilleidae	*Ophryotrocha globopalpata*	0	662	0	1764	0	0	0	0	42
Polychaeta	Orbiniidae	*Scoloplos* sp	0	8	0	28	0	0	0	0	0
Polychaeta	Phyllodocidae	*Protomystides verenae*	0	0	0	0	0	0	12	0	20
Polychaeta	Siboglinidae	*Ridgeia piscesae*	0	0	4	0	0	59	0	0	10
Polychaeta	Syllidae	*Sphaerosyllis cf ridgensis*	0	325	0	12	0	0	3	0	0
Polychaeta	Polychaete unk	non ID cf hesionid	0	0	0	0	0	0	4	0	0
Nematoda	Monhysteridae	*cf Thalassomonhystera*	0	812	0	504	0	0	204	0	2840
Nematoda	unk	unknown sp	4	56	0	12	0	0	0	0	0
Nematoda	cf Chromadoridae	*cf Neochromadora*	0	376	0	316	0	0	620	0	0
Nemertea	Palaeonemertea	unknown sp	0	3632	0	4608	8	0	1252	0	0
		**Justified to full sample**	10308	11024	49368	18626	55136	15750	5399	54912	13923
		**Original numbers assessed**	2577	2756	12342	4657	13784	788	1350	3295	1392
		**# spp present**	5	18	6	17	4	4	17	1	8

Supplementary Table 3. Individual sample residual values from pairwise Procrustes analyses. Pairs in **BOLD** showed a significant relationship ($p<0.05$). Green=low residual values, Red=high residual values (relative color scaling is within rows). ND=no data, Macro=macrofauna, Meio=meiofauna, Bact=bacteria, Arch=archaea, Euk=microeukarya, Micro=bacteria, archaea, and microeukarya proportionally combined based on qPCR.

Temperature (°C)	3.4	5.2	13.4	21.1	27.4	31	33.2	37.1	37.4
	EMw3	ECw11	EMw5	MVw13	MVw12	ECw10	EMw1	EMw4	EMw8
**Macro-Meio**	0.08	0.08	0.08	0.04	0.46	0.10	0.10	0.10	0.10
**Bact-Euk**	0.21	0.32	0.17	0.11	0.17	0.20	0.16	0.18	0.27
Macro-Bact	0.30	0.20	0.07	0.62	0.15	0.18	0.48	0.12	0.10
**Meio-Bact**	0.14	0.27	0.22	0.33	0.47	0.27	0.48	0.13	0.10
Meio-Euk	0.12	0.43	0.12	0.35	0.28	0.28	0.63	0.28	0.28
Macro-Euk	0.06	0.40	0.06	0.32	0.67	0.22	0.70	0.22	0.22
Macro-Arch	0.41	ND	0.41	0.42	0.38	0.24	0.24	0.23	0.24
Meio-Arch	0.58	ND	0.58	0.56	0.20	0.20	0.20	0.20	0.20
Arch-Euk	0.27	ND	0.27	0.80	0.27	0.27	0.80	0.27	0.27
Bact-Arch	0.47	ND	0.45	0.74	0.46	0.15	0.50	0.21	0.28
Micro-Macro	0.44	0.45	0.44	0.51	0.51	0.10	0.10	0.10	0.10
Micro-Meio	0.41	0.54	0.41	0.50	0.13	0.13	0.13	0.13	0.13
Mean	0.29	0.34	0.27	0.44	0.35	0.20	0.38	0.18	0.19
Std deviation	0.16	0.14	0.17	0.22	0.16	0.06	0.24	0.06	0.08

Supplementary Table 4. OTUs and species identified as relatively enriched by ALDEx2 in at least one sample type. OTUs reported here are from the q-PCR balanced microbial community and are preceded by a letter indicating the microbial domain (A=Archaea, B=Bacteria, E=Microeukarya). Associations with highT or lowT clusters in co-occurrence network (Figure 3) are listed on the right.

		Absolute Effect Values (ALDEx2) distinguishing X from Y				
**OTU no. or size class**	**Taxonomy of enriched species or OTUs**	HighT from diffuse	HighT from LowT	LowT from diffuse	LowT from highT	**Cytoscape cluster**
Macrofauna	*Depressigyra globulus* (Gastropoda)		1.34			HighT 1
	Amphisamytha caldarei (Polychaeta, Ampharetidae)				1.94	LowT
	Branchinotogluma tunnicliffeae (Polychaeta, Polynoidae)		1.09			HighT 2
	Paralvinella palmiformis (Polychaeta, Alvinellidae)		1.43			HighT 1
	Paralvinella pandorae (Polychaeta, Alvinellidae)		1.28			HighT 1
	Paralvinella sulfincola (Polychaeta, Alvinellidae)		2.09			HighT 1
	Helicoradomenia juani (Solenogastres)				1.54	LowT
Meiofauna	*Benthoxynus spiculifer* (Hexanauplia, Dirivultidae)		1.99			HighT 1
	Harpacticoid spp. (Hexanauplia, Harpacticoida)				1.75	LowT
	Stygiopontius quadrispinosus (Hexanauplia, Dirivultidae)		1.63			
	cf. *Thalassomonhystera* (Nematoda)				1.71	LowT
	juvenile *Lepetodrilus fucensis* (Gastropoda)				1.41	LowT
	juvenile *Amphisamytha caldarei* (Polychaeta, Ampharetidae)				1.79	LowT
	juvenile *Paralvinella palmiformis* (Polychaeta, Alvinellidae)		1.12			
A_OTU32	Archaea;Asgardaeota		1.47			HighT 2
B_OTU419	Bacteria		1.84			
B_OTU487	Bacteria			1.52	1.43	
B_OTU528	Bacteria			2.16	1.77	
B_OTU759	Bacteria			2.05	1.69	
B_OTU1586	Bacteria				1.45	
B_OTU387	Actinobacteria;Sva0996_marine_group			1.88	1.49	LowT
B_OTU295	Bacteroidetes;Bacteroidia			2.55	1.58	HighT 2
B_OTU506	Bacteroidetes;Bacteroidales BD2-2	1.80	1.81			HighT 2
B_OTU90	Bacteroidetes;Bacteroidales;Marinilabiliaceae; Carboxylicivirga	1.96				HighT 1/2
B_OTU130	Bacteroidetes;Bacteroidales;Marinilabiliaceae; Carboxylicivirga	2.27				HighT 1/2
B_OTU192	Bacteroidetes;Bacteroidales;Marinilabiliaceae; Carboxylicivirga	1.84				HighT 1/2
B_OTU292	Bacteroidetes;Bacteroidales;Marinilabiliaceae; Carboxylicivirga		1.59			HighT 1/2
B_OTU95	Bacteroidetes;VC2.1_Bac22	2.46	2.32			HighT 2
B_OTU268	Bacteroidetes;VC2.1_Bac22		1.64			HighT 2
B_OTU564	Bacteroidetes;VC2.1_Bac22		1.46			HighT 2
B_OTU165	Bacteroidetes;Cytophagales;Cyclobacteriaceae			1.77		
B_OTU271	Bacteroidetes;Cytophagales;Cyclobacteriaceae			1.47		
B_OTU121	Bacteroidetes;Cytophagales;Cyclobacteriaceae; Fulvivirga			2.03		
B_OTU860	Bacteroidetes;Cytophagales;Cyclobacteriaceae; Reichenbachiella			1.92		
B_OTU1724	Bacteroidetes;Flavobacteriales			1.45		
B_OTU246	Bacteroidetes;Flavobacteriales;Crocinitomicaceae			2.54	2.36	
B_OTU285	Bacteroidetes;Flavobacteriales;Cryomorphaceae			1.45		
B_OTU47	Bacteroidetes;Flavobacteriales;Flavobacteriaceae			1.95	1.90	
B_OTU59	Bacteroidetes;Flavobacteriales;Flavobacteriaceae			1.37	1.50	
B_OTU346	Bacteroidetes;Flavobacteriales;Flavobacteriaceae			2.17	1.37	
B_OTU434	Bacteroidetes;Flavobacteriales;Flavobacteriaceae			1.30		
B_OTU520	Bacteroidetes;Flavobacteriales;Flavobacteriaceae		1.59			
B_OTU635	Bacteroidetes;Flavobacteriales;Flavobacteriaceae			1.63		
B_OTU764	Bacteroidetes;Flavobacteriales;Flavobacteriaceae	1.83	1.66			
B_OTU547	Bacteroidetes;Flavobacteriales;Flavobacteriaceae; Euzebyella			2.28	1.65	LowT
B_OTU791	Bacteroidetes;Flavobacteriales;Flavobacteriaceae; Maribacter			1.85		
B_OTU482	Bacteroidetes;Flavobacteriales;Flavobacteriaceae; Maritimimonas		1.83			HighT 2
B_OTU133	Bacteroidetes;Flavobacteriales;Ichthyobacteriaceae; Ichthyobacterium		2.63			HighT 1
B_OTU308	Bacteroidetes;Flavobacteriales;Ichthyobacteriaceae; Ichthyobacterium		2.12			HighT 1
B_OTU97	Bacteroidetes;Sphingobacteriales	1.75	2.13			HighT 2
B_OTU45	Bacteroidetes;Sphingobacteriales;Lentimicrobiaceae		1.82			HighT 2
B_OTU93	Bacteroidetes;Sphingobacteriales;Lentimicrobiaceae		2.21			HighT 2
B_OTU153	Bacteroidetes;Ignavibacteria	3.00				
B_OTU551	Cyanobacteria;Sericytochromatia			2.63	1.83	LowT
B_OTU81	Epsilonbacteraeota;Campylobacteria;Campylobacterales		1.68			HighT 1
B_OTU267	Epsilonbacteraeota;Campylobacterales		1.43			HighT 1
B_OTU444	Epsilonbacteraeota;Campylobacterales		1.80			HighT 1
B_OTU474	Epsilonbacteraeota;Campylobacterales		1.69			HighT 1
B_OTU768	Epsilonbacteraeota;Campylobacterales		1.68			HighT 1
B_OTU834	Epsilonbacteraeota;Campylobacterales		1.84			HighT 1
B_OTU1039	Epsilonbacteraeota;Campylobacterales		1.51			HighT 1

OTU no. or size class	Taxonomy of enriched species or OTUs	HighT from diffuse	HighT from LowT	LowT from diffuse	LowT from highT	Cytoscape cluster
B_OTU2	Epsilonbacteraeota;Campylobacterales;Arcobacteraceae; Arcobacter	2.06	2.91			HighT 1
B_OTU77	Epsilonbacteraeota;Campylobacterales;Arcobacteraceae; Arcobacter		1.73			HighT 1
B_OTU138	Epsilonbacteraeota;Campylobacterales;Arcobacteraceae; Arcobacter		1.71			HighT 1
B_OTU141	Epsilonbacteraeota;Campylobacterales;Arcobacteraceae; Arcobacter		1.80			HighT 1
B_OTU490	Epsilonbacteraeota;Campylobacterales;Arcobacteraceae; Arcobacter	1.51	2.16			HighT 1
B_OTU618	Epsilonbacteraeota;Campylobacterales;Arcobacteraceae; Arcobacter		1.62			HighT 1
B_OTU852	Epsilonbacteraeota;Campylobacterales;Arcobacteraceae; Arcobacter		1.78			HighT 1
B_OTU1341	Epsilonbacteraeota;Campylobacterales;Arcobacteraceae; Arcobacter		1.72			HighT 1
B_OTU158	Epsilonbacteraeota;Campylobacterales;Nitratiruptoraceae; Hydrogenimonas		1.45			HighT 2
B_OTU143	Epsilonbacteraeota;Campylobacterales;Sulfurovaceae; Nitratifractor		1.84			HighT 1
B_OTU337	Epsilonbacteraeota;Campylobacterales;Sulfurovaceae; Nitratifractor		1.78			HighT 1
B_OTU368	Epsilonbacteraeota;Campylobacterales;Sulfurovaceae; Nitratifractor		1.70			HighT 1
B_OTU664	Epsilonbacteraeota;Campylobacterales;Sulfurovaceae; Nitratifractor	2.09	1.84			HighT 1
B_OTU1485	Epsilonbacteraeota;Campylobacterales;Sulfurovaceae; Nitratifractor		1.73			HighT 1
B_OTU198	Epsilonbacteraeota;Campylobacterales;Sulfurovaceae; Sulfurovum		1.75			
B_OTU298	Epsilonbacteraeota;Campylobacterales;Sulfurovaceae; Sulfurovum		1.73			
B_OTU300	Epsilonbacteraeota;Campylobacterales;Sulfurovaceae; Sulfurovum				1.58	
B_OTU1414	Epsilonbacteraeota;Campylobacterales;Sulfurovaceae; Sulfurovum		1.41			
B_OTU1181	Epsilonbacteraeota;Campylobacterales;Thiovulaceae; Sulfurimonas				1.54	
B_OTU42	Patescibacteria;Gracilibacteria	1.78	2.57			
B_OTU53	Patescibacteria;Gracilibacteria				1.55	
B_OTU128	Patescibacteria;Gracilibacteria		2.01			
B_OTU137	Patescibacteria;Gracilibacteria				1.57	
B_OTU149	Patescibacteria;Gracilibacteria		2.16			
B_OTU219	Patescibacteria;Gracilibacteria		1.88			
B_OTU265	Patescibacteria;Gracilibacteria		1.70			
B_OTU356	Patescibacteria;Gracilibacteria		1.68			
B_OTU960	Patescibacteria;Gracilibacteria			1.44		
B_OTU1499	Patescibacteria;Gracilibacteria			1.45		
B_OTU849	Patescibacteria;Gracilibacteria;Absconditabacteriales	1.59	1.81			
B_OTU1804	Patescibacteria;Gracilibacteria;Absconditabacteriales			1.43		
B_OTU44	Alphaproteobacteria			1.89		
B_OTU129	Alphaproteobacteria			2.04	1.39	
B_OTU172	Alphaproteobacteria			1.83		
B_OTU196	Alphaproteobacteria			2.26		
B_OTU291	Alphaproteobacteria			1.68		
B_OTU347	Alphaproteobacteria			1.43		
B_OTU365	Alphaproteobacteria			2.24		
B_OTU416	Alphaproteobacteria			2.46	1.43	
B_OTU455	Alphaproteobacteria			1.52	1.36	
B_OTU574	Alphaproteobacteria			1.58	1.37	
B_OTU763	Alphaproteobacteria			2.29	1.58	
B_OTU915	Alphaproteobacteria			1.76		
B_OTU923	Alphaproteobacteria			1.47	1.63	
B_OTU1063	Alphaproteobacteria			1.42		
B_OTU1575	Alphaproteobacteria			1.34		
B_OTU1876	Alphaproteobacteria			1.58		
B_OTU151	Alphaproteobacteria;Caulobacterales;Hyphomonadaceae; Euryhalocaulis			2.14		
B_OTU456	Alphaproteobacteria;Caulobacterales;Hyphomonadaceae; Euryhalocaulis			1.78		
B_OTU100	Alphaproteobacteria;Caulobacterales;Hyphomonadaceae; Robiginitomaculum			1.85	1.40	
B_OTU181	Alphaproteobacteria;Caulobacterales;Hyphomonadaceae; Robiginitomaculum			2.17	1.83	
B_OTU190	Alphaproteobacteria;Caulobacterales;Hyphomonadaceae; Robiginitomaculum			1.95		
B_OTU280	Alphaproteobacteria;Caulobacterales;Hyphomonadaceae; Robiginitomaculum			2.29	1.41	
B_OTU672	Alphaproteobacteria;Caulobacterales;Hyphomonadaceae; Robiginitomaculum			1.65		
B_OTU703	Alphaproteobacteria;Caulobacterales;Hyphomonadaceae; Robiginitomaculum			1.78		
B_OTU332	Alphaproteobacteria;Caulobacterales;Parvularculaceae			1.65		
B_OTU40	Alphaproteobacteria;Kordiimonadales			1.69		
B_OTU329	Alphaproteobacteria;Kordiimonadales			1.59		
B_OTU478	Alphaproteobacteria;Micavibrionales;Micavibrionaceae			1.46	1.70	

OTU no. or size class	Taxonomy of enriched species or OTUs	HighT from diffuse	HighT from LowT	LowT from diffuse	LowT from highT	Cytoscape cluster
B_OTU117	Alphaproteobacteria;Parvibaculales;PS1_clade			2.39	1.65	
B_OTU156	Alphaproteobacteria;Parvibaculales;PS1_clade			1.41	1.70	
B_OTU173	Alphaproteobacteria;Parvibaculales;PS1_clade			1.70	1.91	
B_OTU266	Alphaproteobacteria;Parvibaculales;PS1_clade			1.90	1.66	
B_OTU283	Alphaproteobacteria;Parvibaculales;PS1_clade			2.15	1.52	
B_OTU49	Alphaproteobacteria;Rhizobiales;Devosiaceae			2.23	1.76	LowT
B_OTU783	Alphaproteobacteria;Rhizobiales;Devosiaceae			1.37	1.61	LowT
B_OTU65	Alphaproteobacteria;Rhizobiales;Rhizobiaceae; Pseudahrensia			1.76		
B_OTU115	Alphaproteobacteria;Rhizobiales;Rhizobiaceae; Pseudahrensia			4.12	1.78	
B_OTU256	Alphaproteobacteria;Rhizobiales;Rhizobiaceae; Pseudahrensia			1.63	1.50	
B_OTU525	Alphaproteobacteria;Rhizobiales;Rhizobiaceae; Pseudahrensia			2.64	1.70	
B_OTU10	Alphaproteobacteria;Rhodobacterales;Rhodobacteraceae			1.91	1.95	
B_OTU32	Alphaproteobacteria;Rhodobacterales;Rhodobacteraceae			1.92	1.69	
B_OTU75	Alphaproteobacteria;Rhodobacterales;Rhodobacteraceae			1.72		
B_OTU131	Alphaproteobacteria;Rhodobacterales;Rhodobacteraceae			1.48	1.74	
B_OTU261	Alphaproteobacteria;Rhodobacterales;Rhodobacteraceae			1.87		
B_OTU357	Alphaproteobacteria;Rhodobacterales;Rhodobacteraceae			2.27		
B_OTU372	Alphaproteobacteria;Rhodobacterales;Rhodobacteraceae			1.91		
B_OTU603	Alphaproteobacteria;Rhodobacterales;Rhodobacteraceae			1.54	2.32	
B_OTU616	Alphaproteobacteria;Rhodobacterales;Rhodobacteraceae			1.55	1.43	
B_OTU730	Alphaproteobacteria;Rhodobacterales;Rhodobacteraceae			2.26	2.13	
B_OTU1038	Alphaproteobacteria;Rhodobacterales;Rhodobacteraceae			1.65	1.55	
B_OTU1129	Alphaproteobacteria;Rhodobacterales;Rhodobacteraceae			1.33		
B_OTU1137	Alphaproteobacteria;Rhodobacterales;Rhodobacteraceae			1.51		
B_OTU29	Alphaproteobacteria;Rhodobacterales;Rhodobacteraceae;Roseobacter_clade_NAC11-7			2.80	2.40	
B_OTU104	Alphaproteobacteria;Rhodobacterales;Rhodobacteraceae;Roseobacter_clade_NAC11-7			2.31		
B_OTU436	Alphaproteobacteria;Rhodobacterales;Rhodobacteraceae;Roseobacter_clade_NAC11-7			1.93	2.11	
B_OTU484	Alphaproteobacteria;Rhodobacterales;Rhodobacteraceae;Roseobacter_clade_NAC11-7			1.31		
B_OTU522	Alphaproteobacteria;Rhodobacterales;Rhodobacteraceae;Roseobacter_clade_NAC11-7			1.81	2.21	
B_OTU971	Alphaproteobacteria;Rhodobacterales;Rhodobacteraceae;Roseobacter_clade_NAC11-7			1.60		
B_OTU972	Alphaproteobacteria;Rhodobacterales;Rhodobacteraceae;Roseobacter_clade_NAC11-7			1.50	1.50	
B_OTU1086	Alphaproteobacteria;Rhodobacterales;Rhodobacteraceae;Roseobacter_clade_NAC11-7			1.80		
B_OTU125	Alphaproteobacteria;Rhodobacterales;Rhodobacteraceae; Sedimentitalea			1.44		
B_OTU146	Alphaproteobacteria;Rhodobacterales;Rhodobacteraceae; Sedimentitalea			2.73	2.07	
B_OTU259	Alphaproteobacteria;Rhodobacterales;Rhodobacteraceae; Sedimentitalea			1.67	1.97	
B_OTU435	Alphaproteobacteria;Rhodobacterales;Rhodobacteraceae; Sedimentitalea			1.68	1.68	
B_OTU964	Alphaproteobacteria;Rhodobacterales;Rhodobacteraceae; Sedimentitalea			2.13	1.66	
B_OTU1773	Alphaproteobacteria;Rhodobacterales;Rhodobacteraceae; Sedimentitalea			1.41	1.57	
B_OTU134	Alphaproteobacteria;Rhodovibrionales;Kiloniellaceae			2.04		
B_OTU373	Alphaproteobacteria;Rickettsiales			2.00	1.66	
B_OTU418	Alphaproteobacteria;Rickettsiales			1.50		
B_OTU200	Deltaproteobacteria		1.68			
B_OTU575	Deltaproteobacteria			1.88		
B_OTU803	Deltaproteobacteria				1.49	
B_OTU666	Deltaproteobacteria;Bdellovibrionales;Bdellovibrionaceae;OM27_clade			1.74		
B_OTU235	Deltaproteobacteria;Desulfobacterales;Desulfobulbaceae		1.48			HighT 2
B_OTU607	Deltaproteobacteria;Desulfobacterales;Desulfobulbaceae		1.44			HighT 2
B_OTU343	Gammaproteobacteria			2.19	1.94	
B_OTU87	Gammaproteobacteria;Cellvibrionales;Halieaceae			1.65		
B_OTU684	Gammaproteobacteria;Cellvibrionales;Halieaceae			1.62		
B_OTU160	Gammaproteobacteria;Cellvibrionales;Halieaceae; Halioglobus			2.09	2.06	LowT
B_OTU14	Gammaproteobacteria;Cellvibrionales;Spongiibacteraceae; Dasania			2.46		LowT
B_OTU36	Gammaproteobacteria;Nitrosococcales;Methylophagaceae;Marine_Methylotrophic_Grp_3			2.06		
B_OTU69	Gammaproteobacteria;Nitrosococcales;Methylophagaceae;Marine_Methylotrophic_Grp_3			2.94	2.04	
B_OTU82	Gammaproteobacteria;Nitrosococcales;Methylophagaceae;Marine_Methylotrophic_Grp_3			1.93		
B_OTU399	Gammaproteobacteria;Nitrosococcales;Methylophagaceae;Marine_Methylotrophic_Grp_3			1.84	1.63	
B_OTU388	Gammaproteobacteria;Thiotrichales;Thiotrichaceae; Cocleimonas			1.70		
B_OTU277	WPS-2 (Candidate Phylum Eremiobacteraeota)			1.77		
E_OTU52	Amoebozoa;Lobosa;Tubulinea;Leptomyxida;Flabellulidae			1.57		
E_OTU55	Amoebozoa;Lobosa;Tubulinea;Leptomyxida;Flabellulidae			1.65		
E_OTU94	Amoebozoa;Lobosa;Tubulinea;Leptomyxida;Rhizamoeba; R. saxonica				1.48	
E_OTU18	Excavata;Discoba;Jakobida	2.10				HighT 1/2
E_OTU3	Opisthokonta;Fungi;Ascomycota;Pezizomycotina	2.15				HighT 2

Supplementary Table 5. Microbial taxa with weak associations to highT or lowT grab samples, indicated by intermediate-sized nodes in network diagram (Figure 3).

			Covariance (proportionality)			Relative enrichment in X vs. Y †			
Supergroup/Phylum/ Class	Microbial taxa ‡	*Cytoscape cluster	Total no. positive ρ values	No. positive ρ with macro/meio	Average ρ with macro/meio	HighT vs. LowT grabs	HighT grabs vs. HighT diffuse)	LowT vs. HighT grabs	LowT grabs vs. LowT diffuse
Covariance with a single faunal species									
Crenarchaeota	A_or_Desulfurococcales	HighT 2	14	1	0.84				
Euryarchaeota	A_cl_Thermoplasmata	HighT 1	27	1	0.76				
Euryarchaeota	A_cl_Thermoplasmata Marine Group III	HighT 1	11	1	0.78				
Euryarchaeota	A_Methanocorpusculum	HighT 2	7	1	0.76				
Euryarchaeota	A_Pyrococcus	HighT 2	28	1	0.81				
Nanoarchaeota	A_cl_Nanohaloarchaeia DSEG	HighT 2	28	1	0.82				
Nanoarchaeota	A_cl_Woesearchaeia	HighT 1	5	1	0.95				
Acidobacteria	B_fa_Thermoanaerobaculaceae Subgroup 23	HighT 2	22	1	0.78				
Actinobacteria	B_fa_Microtrichaceae	LowT	27	1	0.77				
Alphaproteobacteria	B_Methylobacterium	HighT 1	4	1	0.75				
Bacteroidetes	B_cl_Bacteroidia	HighT 2	31	1	0.77			X	X
Bacteroidetes	B_ph_Bacteroidetes	HighT 2	29	1	0.85				
Calditrichaeota	B_Caldithrix	HighT 2	32	1	0.81				
Chloroflexi	B_cl_Anaerolineae	HighT 2	23	1	0.81				
Deltaproteobacteria	B_Desulfuromusa	HighT 2	27	1	0.85				
Epsilonbacteraeota	B_cl_Campylobacteria	HighT 1	26	1	0.77				
Epsilonbacteraeota	B_fa_Helicobacteraceae	HighT 1	2	1	0.78				
Epsilonbacteraeota	B_fa_Nautiliaceae	HighT 2	24	1	0.80				
Epsilonbacteraeota	B_fa_Sulfurovaceae	HighT 2	45	1	0.77				
Epsilonbacteraeota	B_Thiofractor	HighT 2	29	1	0.79				
Firmicutes	B_fa_Ruminococcaceae	HighT 2	40	1	0.86				
Nitrospinae	B_fa_Nitrospinaceae	LowT	31	1	0.78				
	B_sg_Patescibacteria	HighT 1	27	1	0.77				
Amoebozoa	E_cl_Breviatea NAMAKO-1	HighT 1	8	1	0.76				
Archaeplastida	E_Prasinoderma singularis	HighT 2	6	1	0.76				
Cercozoa	E_cl_Filosa/Imbricatea	HighT 2	3	1	0.76				
Ciliophora	E_fa_Vaginicolidae	LowT	10	1	0.78				
Dinoflagellata	E_or_Dino Group I Clade 2	HighT 2	7	1	0.77				
Enrichment only									
	B_do_Bacteria		4	0	-	X		X	X
Alphaproteobacteria	B_cl_Alphaproteobacteria		24	0	-			X	X
Alphaproteobacteria	B_Euryhalocaulis		19	0	-				X
Alphaproteobacteria	B_Robiginitomaculum		23	0	-			X	X
Alphaproteobacteria	B_fa_Parvularculaceae		33	0	-				X
Alphaproteobacteria	B_or_Kordiimonadales		12	0	-				X
Alphaproteobacteria	B_or_Micavibrionales		21	0	-				X
Alphaproteobacteria	B_fa_Micavibrionaceae		13	0	-			X	
Alphaproteobacteria	B_or_Parvibaculales PS1 clade		17	0	-			X	X
Alphaproteobacteria	B_Pseudahrensia		18	0	-			X	X
Alphaproteobacteria	B_fa_Rhodobacteraceae		16	0	-			X	X
Alphaproteobacteria	B_fa_Rhodobacteraceae NAC11-7 clade		8	0	-			X	X
Alphaproteobacteria	B_Sedimentitalea		20	0	-			X	X
Alphaproteobacteria	B_fa_Kiloniellaceae		20	0	-				X
Alphaproteobacteria	B_or_Rickettsiales		15	0	-			X	X
Bacteroidetes	B_Fulvivirga		17	0	-				X
Bacteroidetes	B_Reichenbachiella		20	0	-				X
Bacteroidetes	B_fa_Crocinitomicaceae		3	0	-			X	X
Bacteroidetes	B_fa_Cryomorphaceae		11	0	-				X
Bacteroidetes	B_fa_Flavobacteriaceae		6	0	-	X	X	X	X
Bacteroidetes	B_Maribacter		22	0	-				X
Cand. Eremiobacteraeota	B_cand_WPS-2		21	0	-				X
Cand. Gracilibacteria	B_cl_Gracilibacteria		3	0	-	X	X	X	X
Cand. Gracilibacteria	B_or_Absconditabacteriales		8	0	-	X	X		X
Deltaproteobacteria	B_cl_Deltaproteobacteria		2	0	-	X		X	X
Epsilonbacteraeota	B_Sulfurovum		7	0	-	X		X	
Epsilonbacteraeota	B_Sulfurimonas		7	0	-			X	
Gammaproteobacteria	B_fa_Halieaceae		23	0	-				X
Gammaproteobacteria	B_cl_Gammaproteobacteria		21	0	-			X	X
Gammaproteobacteria	B_fa_Methylophagaceae Marine Methylotrophic Grp3		13	0	-			X	X
Gammaproteobacteria	B_Cocleimonas		28	0	-				X
Ignavibacteriae	B_cl_Ignavibacteria		12	0	-		X		
Amoebozoa	E_fa_Flabellulidae		9	0	-				X
Amoebozoa	E_Rhizamoeba saxonica		1	0	-				X

‡ Multiple OTUs binned by identical taxonomic assignment. Taxa are preceded by single letter code indicating microbial domain (A=Archaea, B=Bacteria, E=Microeukarya) and two letter code indicating taxonomic depth of classification, if other than genus (sg=supergroup, do=domain, ph=phylum, cl=class, or=order, fa=family).

* Cluster association in network figure 3.

† Relative enrichment determined by ALDEx2 pairwise tests.
